# Endothelial Piezo1 sustains muscle capillary density and contributes to physical activity

**DOI:** 10.1172/JCI141775

**Published:** 2022-03-01

**Authors:** Fiona Bartoli, Marjolaine Debant, Eulashini Chuntharpursat-Bon, Elizabeth L. Evans, Katie E. Musialowski, Gregory Parsonage, Lara C. Morley, T. Simon Futers, Piruthivi Sukumar, T. Scott Bowen, Mark T. Kearney, Laeticia Lichtenstein, Lee D. Roberts, David J. Beech

**Affiliations:** 1 School of Medicine and; 2 School of Biomedical Sciences, University of Leeds, Leeds, United Kingdom.

**Keywords:** Muscle Biology, Vascular Biology, Ion channels, Microcirculation, Skeletal muscle

## Abstract

Piezo1 forms mechanically activated nonselective cation channels that contribute to endothelial response to fluid flow. Here we reveal an important role in the control of capillary density. Conditional endothelial cell–specific deletion of Piezo1 in adult mice depressed physical performance. Muscle microvascular endothelial cell apoptosis and capillary rarefaction were evident and sufficient to account for the effect on performance. There was selective upregulation of thrombospondin-2 (TSP2), an inducer of endothelial cell apoptosis, with no effect on TSP1, a related important player in muscle physiology. TSP2 was poorly expressed in muscle endothelial cells but robustly expressed in muscle pericytes, in which nitric oxide (NO) repressed the *Tsp2* gene without an effect on *Tsp1*. In endothelial cells, Piezo1 was required for normal expression of endothelial NO synthase. The data suggest an endothelial cell–pericyte partnership of muscle in which endothelial Piezo1 senses blood flow to sustain capillary density and thereby maintain physical capability.

## Graphical abstract

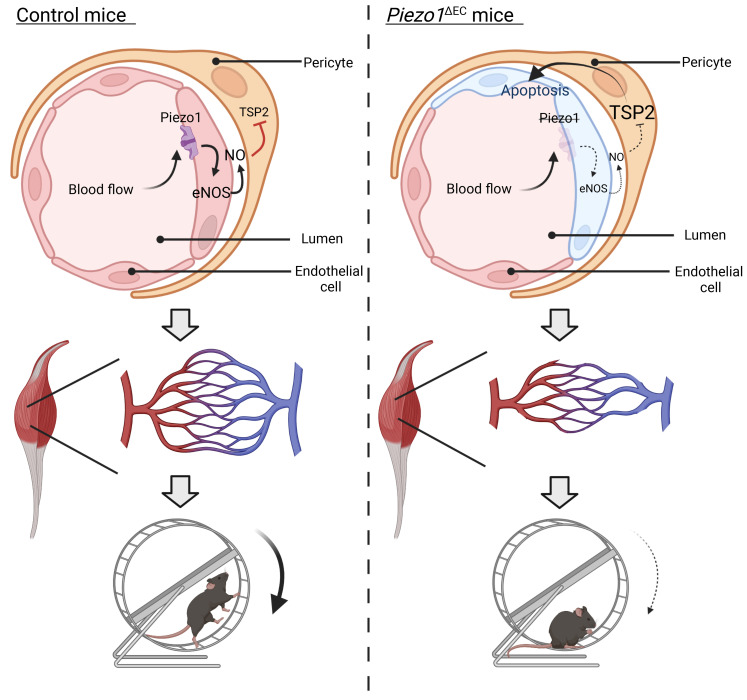

## Introduction

Physical fitness is important for survival. Lack of physical activity results in detraining, lower performance, and ultimately, incapacity. At least 2500 years ago it was first documented that exercise is good for health (1, 2). Since the first epidemiological studies (3), research has continued to bestow on us a wealth of evidence that exercise protects against a host of ailments and life-threatening conditions that affect large numbers of people in 21st-century societies through heart attacks, strokes, diabetes, depression, dementia, cancer, osteoporosis, chronic kidney disease, and sexual dysfunction (4–10). Exercise is commonly encouraged, even prescribed, as therapy. We know that if every human could maintain a suitable level of exercise and fitness, many disease problems would be less severe and quality of life would be better. However, there are major challenges due to illness, injuries, and the intellectual and computational demands of modern societies that increase the prevalence of sedentary lifestyles. The World Health Organization suggests that physical inactivity is the fourth leading cause of death worldwide.

Although many responses to exercise are known, how the benefits of exercise are initially triggered at a molecular level is mysterious. It is reasonable to suppose that there must be a biological system containing a sensor or sensors to indicate to the body how much physical activity is occurring, but the sensor is obscure. Exercise research has suggested that the site of the sensor might be the endothelium and that blood flow or other hemodynamic parameters are sensed (11–13). The endothelium consists of a monolayer of cells lining the inner surface of all blood vessels throughout the body: the cellular interface between blood and tissue and the only structure known to detect the rate of blood flow, something at which it is highly adept (14–16). Physical activity increases the rate of blood flow (11), so the benefits of exercise might originate at least partly in the detection of this increased flow. Therefore, if the sensor of increased flow could be identified there would be an advance in understanding how humans respond to exercise and achieve health benefits.

In order to test the endothelial flow sensor hypothesis, it is advantageous to know the molecular processes of the endothelium that detect blood flow during physical activity. There are many competing ideas about how, in general, vascular flow sensing is mediated (14, 15). We have suggested that the relatively recently discovered Piezo protein, Piezo1, is pivotal (17–19). Piezo1 forms mechanically activated Ca^2+^-permeable nonselective cation channels in endothelial cells (17, 20, 21). These channels are activated by fluid flow (17–19, 22). Previous evidence suggested that Piezo1 was responsible for increased blood pressure in mice undertaking voluntary exercise, suggesting a link to physical activity (19). However, only modest transient reduction in performance was observed (19). In these studies, the protocol involved conditional deletion of endothelial cell Piezo1 in adult mice 2 weeks prior to analysis (19). We reasoned that this might not be sufficient to impact all relevant processes and so we lengthened the period to 10 weeks. This allowed us to observe profound loss of physical performance and an important role for endothelial Piezo1 in muscle capillarity.

## Results

### Unaffected anatomical parameters.

Endothelial cell–specific disruption of Piezo1 was induced in adult mice (*Piezo1^ΔEC^* mice) and major organs were harvested 10 weeks later. As expected, *Piezo1^ΔEC^* mice showed a significant decrease in *Piezo1* mRNA abundance in whole gastrocnemius muscle and heart and most obviously in endothelial cells isolated from skeletal muscle (SkECs) (Supplemental Figure 1, A–C; supplemental material available online with this article; https://doi.org/10.1172/JCI141775DS1), thus validating an efficient depletion of endothelial cell Piezo1 and good purity of SkECs. We previously showed no differences in major organs 2 weeks after the conditional deletion of endothelial cell Piezo1 (19). Similarly, at 10 weeks after deletion we found no differences in skeletal muscle, heart, or liver weights relative to total body weight in *Piezo1^ΔEC^* mice (Supplemental Figure 1D).

### Sustained attenuation of physical activity.

**Figure 1:**
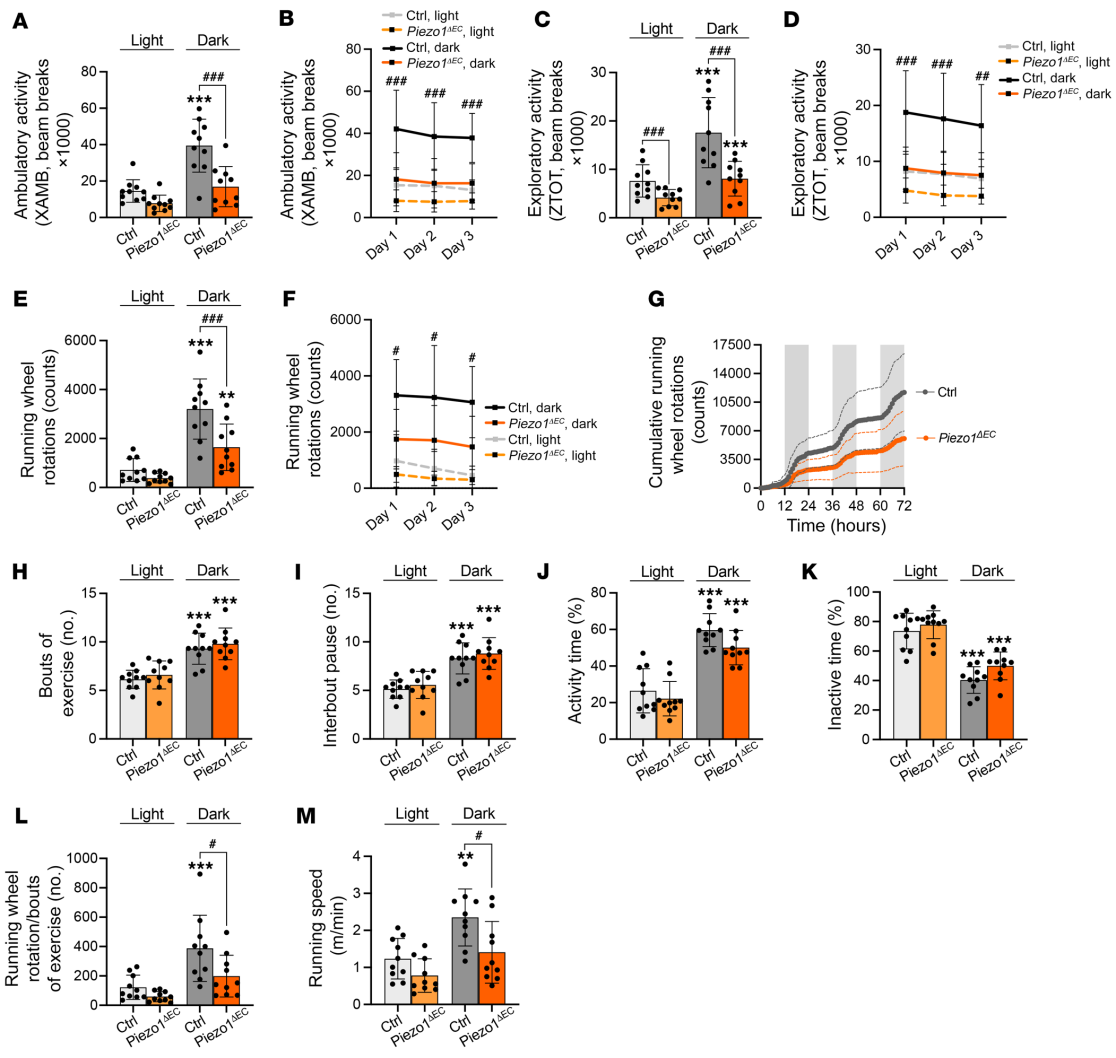
Endothelial Piezo1 determines physical performance but not desire for activity. Throughout the figure, data in gray represent control mice (Ctrl) and data in orange are for *Piezo1^ΔEC^* mice. The lighter color is for data sampled during the light cycle (inactive period) and darker color for data during the dark cycle (active period). (**A**) Pooled and averaged ambulatory activity (XAMB) across 3 light and dark cycles for Ctrl mice and *Piezo1^ΔEC^* mice. (**B**) Day-by-day averaged ambulatory activity. (**C**) Similar to **A** but showing exploratory activity (ZTOT). (**D**) Day-by-day averaged exploratory activity. (**E**) Similar to **A** but showing running wheel rotation counts (voluntary activity). (**F**) Day-by-day averaged voluntary activity. (**G**) Cumulative running wheel rotations during 72-hour recording. Gray shaded areas indicate the dark cycles. (**H**) Number of active bouts of exercise (periods of activity defined as activity seen in 1 or more consecutive 10-minute intervals). (**I**) Number of interbout pauses (periods of inactivity between 2 bouts of exercise). (**J**) Percentage of time for which mice were active on the wheel. (**K**) Percentage of time for which mice were off the wheel (inactive time). (**L**) Normalization of running wheel rotations per active bouts of exercise. (**M**) Running-wheel speed. All data are for *n =* 10 mice per group (mean ± SD). Superimposed dots are the individual underlying data values for each individual mouse. **P < 0.01, ***P < 0.001 vs. light cycle; ^#^*P* < 0.05, ^##^*P* < 0.01, ^###^*P* < 0.001 vs. Ctrl mice. Statistical significance was evaluated using 2-way ANOVA followed by Tukey’s HSD post hoc test for multiple comparisons.

Ten weeks after the disruption of endothelial Piezo1, the physical performance of control and *Piezo1^ΔEC^* mice was analyzed. Compared with matched control mice, *Piezo1^ΔEC^* mice showed striking sustained reductions in ambulatory (Figure 1, A and B), vertical exploratory (Figure 1, C and D), and running wheel activities (Figure 1, E–G). The data suggest an important role for endothelial Piezo1 in sustaining normal physical activity.

### Desire for physical activity is not affected.

A potential explanation for lower activity is decreased psychological interest in exercise. However, there were no differences in the numbers of bouts of activity (Figure 1H) or interbout pauses (Figure 1I) or in the total periods of activity (Figure 1J) or inactivity (Figure 1K). Instead, there were fewer running wheel revolutions per bout of exercise (Figure 1L) and running speed was lower (Figure 1M). The data suggest specific reduction in the ability to perform physically, without less desire to exercise.

### Respiration, energy metabolism, and cardiac function are unchanged.

Potential mechanistic explanations for lower performance are reduced respiration or metabolism. As *Piezo1^ΔEC^* mice had a lower body weight than control mice (Supplemental Figure 2A), they displayed reduced oxygen consumption and tendency to less carbon dioxide production, but these differences were due to a mass effect (Supplemental Figure 2B). No differences were found after analysis of covariance (ANCOVA) (Supplemental Figure 2, B–D). Furthermore, no differences were found in respiratory exchange ratio (Supplemental Figure 2E), energy expenditure (Supplemental Figure 2F), food consumption (Supplemental Figure 2G), or circadian patterns of these parameters (Supplemental Figure 2H and Supplemental Figure 3, A–E). An alternative explanation could be reduced cardiac function and so echocardiography was performed. *Piezo1^ΔEC^* mice showed no signs of cardiac alteration as determined by morphometric and echocardiographic measurements (Supplemental Figure 1D and Supplemental Figure 4, A–M). The data suggest no changes in respiration, metabolism, or cardiac function and thus that these parameters do not mediate the reduced performance.

### Skeletal muscle mass and fiber types are unchanged.

**Figure 2:**
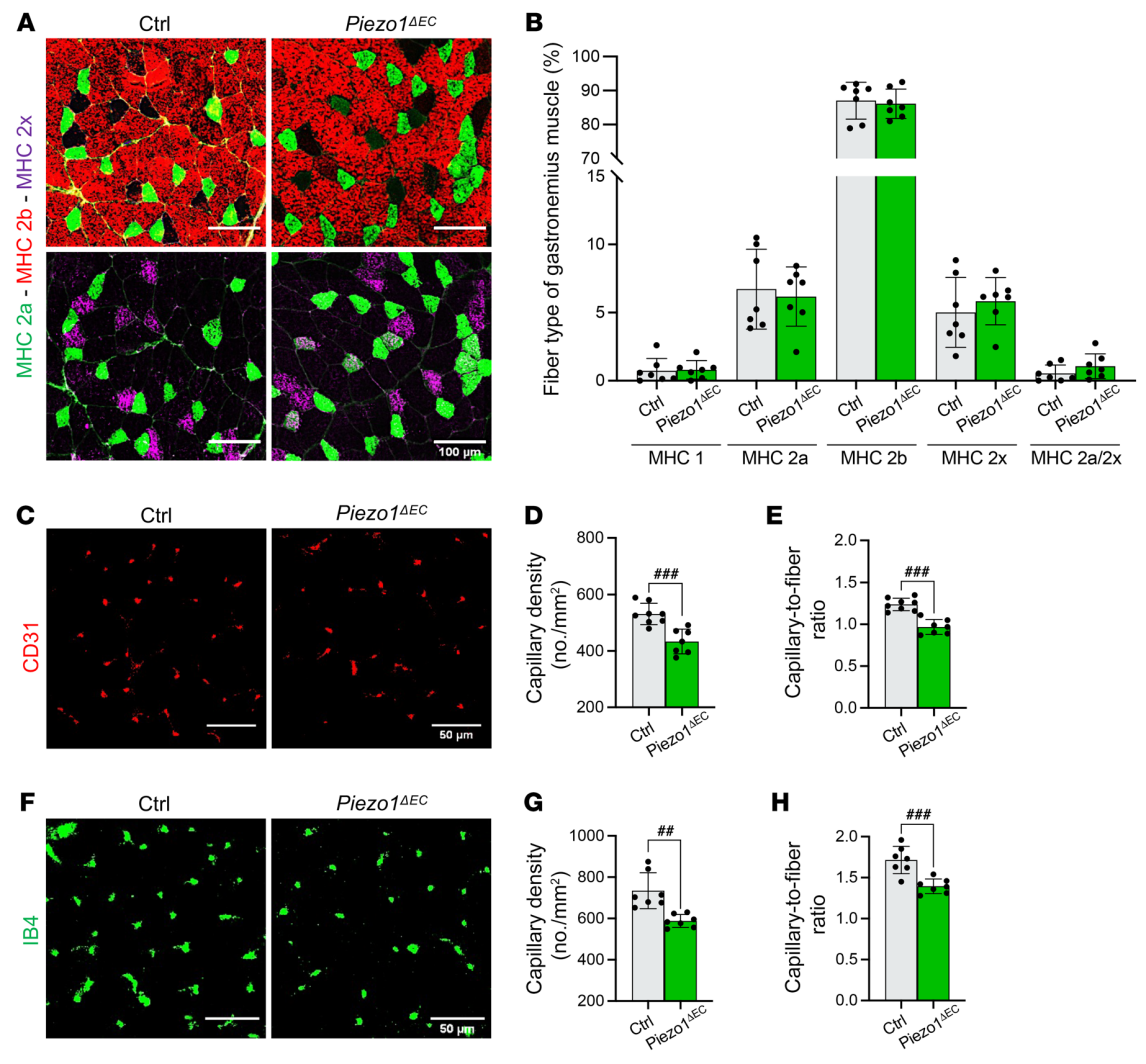
Endothelial Piezo1 specifically affects microvascular density. (**A**) Immunohistochemistry of gastrocnemius muscle cross sections for myosin heavy chain (MHC) type 2a (green) plus type 2b (red, left) or type 2x (magenta, right). Scale bars: 100 μm. (**B**) Quantification of the relative frequency of the different fiber types in gastrocnemius muscle. (**C**) Immunohistochemistry for CD31 (red) to visualize endothelial cells in capillaries of gastrocnemius muscle sections. Scale bars: 50 μm. (**D**) Mean data for capillary density measured from images of the type shown in **C**. (**E**) Similar to **D** but showing mean data for the ratio of capillaries to muscle fibers. (**F**) Immunohistochemistry for isolectin B4 (IB4, green) to visualize endothelial cells in capillaries of gastrocnemius muscle sections. Scale bars: 50 μm. (**G**) Mean data for capillary density measured from images of the type shown in **F**. (**H**) Similar to **G** but showing mean data for the ratio of capillaries to muscle fibers. All data are for *n =* 7 to 8 mice per group (mean ± SD). Superimposed dots are the underlying data values for each individual mouse. Gray indicates muscles from Ctrl mice and green indicates muscles from *Piezo1^ΔEC^* mice. ^##^*P* < 0.01, ^###^*P* < 0.001 vs. ctrl mice. Statistical significance was evaluated using Student’s *t* test.

An alternative explanation could be a changed muscle mass or fiber-type composition. No differences in mass were found in hind limb muscles that contribute to running performance — rectus femoris, vastus lateralis, gastrocnemius, and soleus — in *Piezo1^ΔEC^* mice compared with controls (Supplemental Figure 1D). Fiber type within muscle is important for performance, but there were no differences in the percentages of fiber types 1, 2a, 2b, 2x, or 2a/2x (Figure 2, A and B). In addition, myofiber density and size distribution were investigated by staining with wheat germ agglutinin (WGA) to label cell membranes. Fiber density, size, and distribution were similar between controls and *Piezo1^ΔEC^* mice (Supplemental Figure 5, A–D). Consistent with these observations, gene expression analysis suggested no changes in markers of fibrosis, endoplasmic reticulum stress, fiber growth, fiber switch, hypoxia, inflammation, glucose and lipid metabolism, mitochondrial biogenesis, or immune cell markers (Supplemental Table 1). The data suggest no changes in skeletal muscle mass, fiber type, or gene expression and thus that these parameters do not mediate the reduced performance.

### Lower microvascular density.

Efficient perfusion of skeletal muscle fibers is critical in physical performance to provide appropriate oxygen and nutrients and remove waste products. As such, we analyzed the capillary density by staining muscle sections for the endothelial cell markers CD31/PECAM-1 and isolectin B4 (IB4). Importantly, there were approximately 20% reductions in capillary density and capillary-to-fiber ratio in *Piezo1^ΔEC^* mice (Figure 2, C–H). Prior work has suggested only a small capillary reserve, such that reductions of more than 10% significantly affect physical performance (23). We also analyzed the capillary density on cardiac sections by staining for IB4. Consistent with the lack of effect on cardiac function, fiber density, capillary density, and capillary-to-fiber ratio were not affected in *Piezo1^ΔEC^* mice (Supplemental Figure 5, E–H), suggesting a vascular bed specificity of endothelial Piezo1 contribution to vascular density. The data suggest that impaired physical performance could be explained by reduced skeletal muscle microvascular density.

### Microvascular and endothelial cell regression.

**Figure 3:**
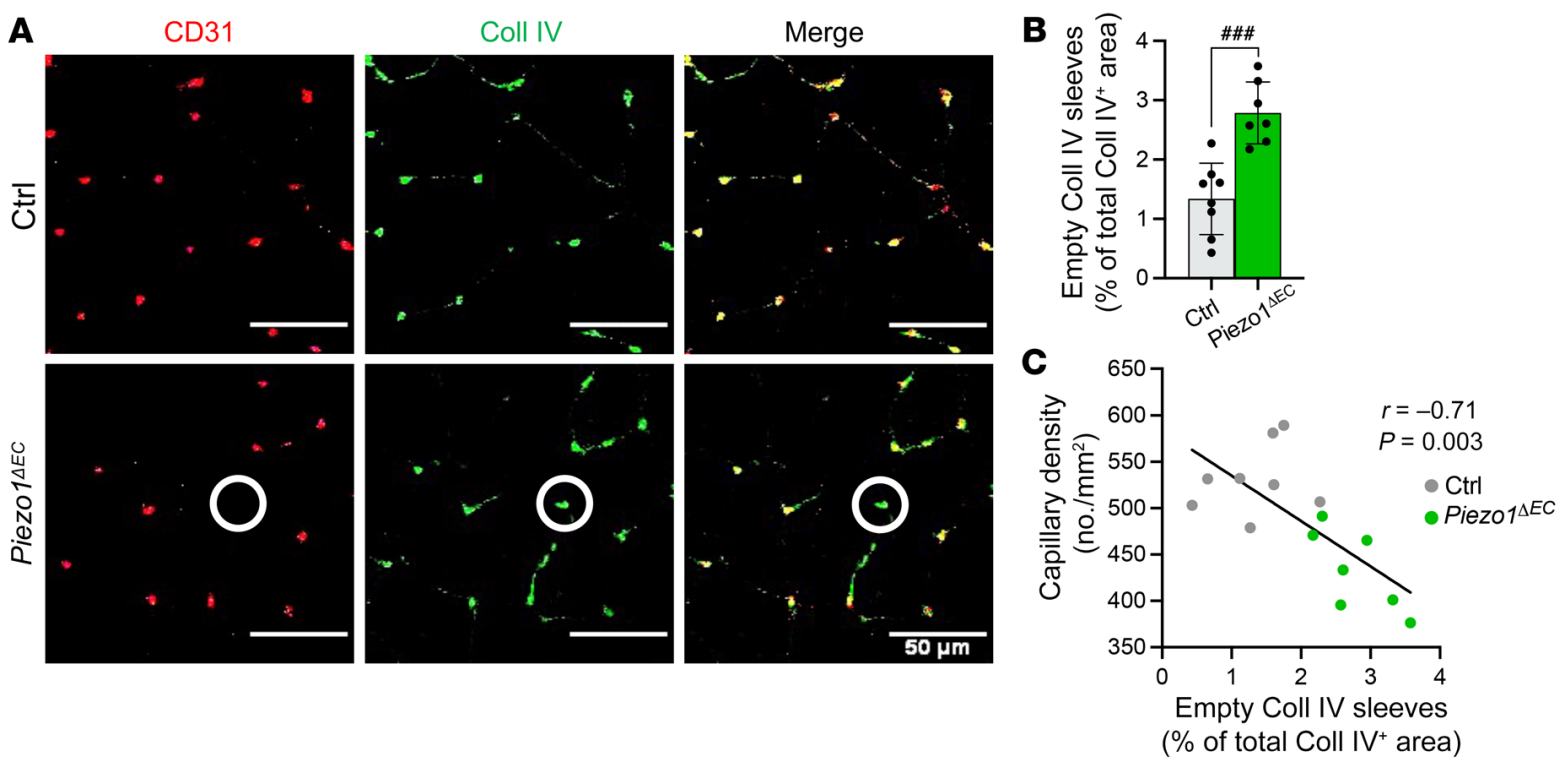
Protection against endothelial microvascular rarefaction. (**A**) Immunohistochemistry for CD31 (red) and type IV collagen (Coll IV, green) to visualize capillaries and basement membrane, respectively, in gastrocnemius muscle sections. Merged images are shown on the right. Scale bars: 50 μm. Superimposed circles highlight an example of a regressing vessel (CD31^–^Coll IV^+^). (**B**) Quantification of empty Coll IV sleeves in gastrocnemius muscle sections, based on images of the type shown in **A**. (**C**) Pearson’s correlation of capillary density and empty Coll IV sleeves (*r* = –0.71, *P =* 0.003). The black line is the correlation fit. All data are for *n =* 7 to 8 mice per group (mean ± SD). Superimposed dots are the underlying data values for each mouse. Gray indicates muscles from Ctrl mice and green indicates muscles from *Piezo1^ΔEC^* mice. ^###^*P* < 0.001 vs. Ctrl mice. Statistical significance was evaluated using Student’s *t* test (**B**) or Pearson’s correlation (*r*) test (**C**).

Piezo1 was conditionally disrupted once mice reached the adult stage and thus when the microvasculature was established, and so we considered that the lower microvascular density might be due to regression of preexisting vessels (i.e., that rarefaction occurred). Rarefaction is characterized by empty sleeves of vascular basement membrane, left behind when endothelial cells disappear. We investigated skeletal muscle by immunostaining for CD31 and type IV collagen (Coll IV), a major component of the basement membrane. *Piezo1^ΔEC^* mice showed more empty Coll IV sleeves (CD31^–^Coll IV^+^; Figure 3, A and B). A significant negative correlation existed between capillary density and number of empty type Coll IV sleeves (Figure 3C). Muscle cross sections (Supplemental Figure 6A) and longitudinal sections (Supplemental Figure 6E) were also stained for NG2 proteoglycan (NG2), a pericyte marker, because these perivascular cells are also implicated in vessel stability (24). *Piezo1^ΔEC^* mice showed a tendency to decreased pericyte density in skeletal muscle (Supplemental Figure 6B) and significantly decreased pericyte-to-fiber ratio (Supplemental Figure 6C). There was a positive correlation between capillary (endothelial cell marker) and pericyte densities (Supplemental Figure 6D) and pericyte coverage of endothelial cells was unchanged (Supplemental Figure 6F). The data suggest that reduced physical performance is due to microvascular regression caused by loss of endothelial cells and pericytes.

### Endothelial cell apoptosis.

**Figure 4:**
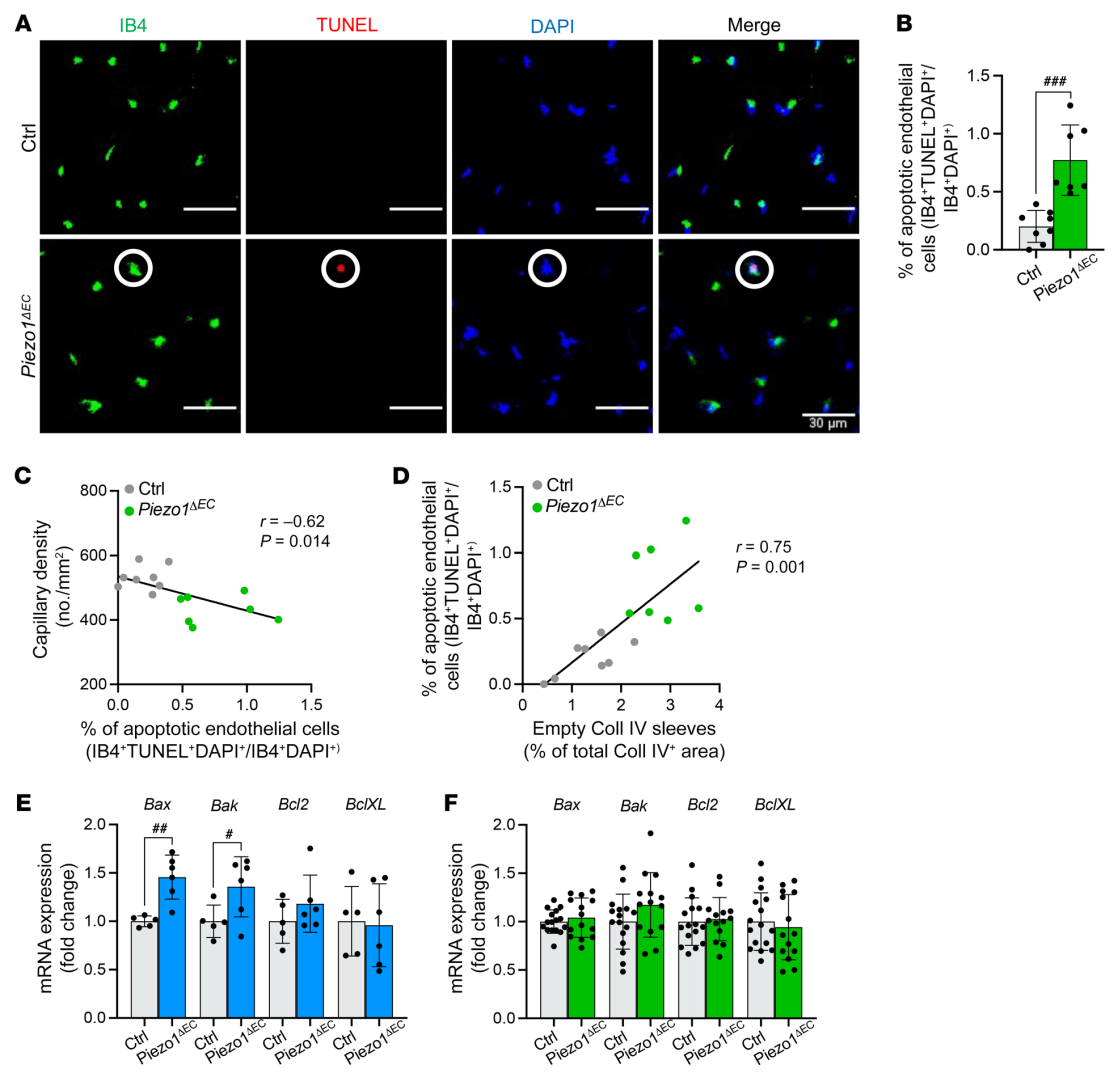
Protection against endothelial cell apoptosis. (**A**) Immunohistochemistry for endothelial cells in capillaries (IB4, green), apoptotic cells (TUNEL, red), and nuclei (DAPI, blue) in gastrocnemius muscle sections. Merged images are shown on the right. Scale bars: 30 μm. Superimposed circles highlight an example of an apoptotic endothelial cell (IB4^+^TUNEL^+^DAPI^+^). (**B**) Quantification of apoptotic endothelial cell percentages in Ctrl and *Piezo1^ΔEC^* gastrocnemius muscle using images of the type shown in **A**. (**C**) Pearson’s correlation analysis of capillary density and percentage of apoptotic endothelial cells (*r* = –0.62, *P =* 0.014). (**D**) Pearson’s correlation analysis of apoptotic endothelial cell percentage and capillary regression (*r* = 0.75, *P =* 0.001). The black lines are the correlation fits. All data are for *n =* 7 to 8 mice per group (mean ± SD). (**E**) Quantitative PCR mRNA expression data for proapoptotic markers (*Bax*, *Bak*) and antiapoptotic markers (*Bcl2*, *BclXL*) in endothelial cells isolated from skeletal muscle of Ctrl (gray) and *Piezo1^ΔEC^* (blue) mice. (**F**) Quantitative PCR mRNA expression data for proapoptotic markers (*Bax*, *Bak*) and antiapoptotic markers (*Bcl2*, *BclXL*) in whole gastrocnemius muscle of Ctrl (gray) and *Piezo1^ΔEC^* (green) mice. RNA abundance was normalized to housekeeping gene expression and is presented as the fold-change relative to that in Ctrl mice. All data are for *n =* 5 to 6 mice per group for endothelial cells and *n =* 14 to 16 mice per group for whole muscle (mean ± SD). Superimposed dots are the individual underlying data values for each mouse. ^#^*P* < 0.05, ^##^*P* < 0.01, ^###^*P* < 0.001 vs. Ctrl mice. Statistical significance was evaluated using Student’s *t* test, except in **C **and **D** where Pearson’s correlation was used.

We surmised that the microvascular regression reflects an organized physiological process mediated by cell apoptosis, possibly originating in endothelial cells. To test this hypothesis, TUNEL assays were performed on muscle sections costained for endothelial cells (IB4 labeling) and cell nuclei (DAPI). There was a striking increase in the percentage of apoptotic IB4-positive cells in *Piezo1^ΔEC^* mice compared with control mice (Figure 4, A and B). There was a significant negative correlation of apoptotic endothelial cells with capillary density and positive correlation with empty Coll IV sleeves (Figure 4, C and D). Furthermore, proapoptotic gene expression markers were significantly upregulated in SkECs isolated from *Piezo1^ΔEC^* mice (Figure 4E). There were no changes in apoptosis markers in whole muscle analysis, consistent with the myocytes being dominant and normal (Figure 4F). IB4 may also bind some immune cells but inflammatory markers were not changed in *Piezo1^ΔEC^* mice (Supplemental Table 1) and the empty Coll IV sleeve data support the suggestion that endothelial cells were the primary apoptotic cell. The data suggest that the microvascular rarefaction is mediated by endothelial cell apoptosis.

### Selective upregulation of thrombospondin-2.

**Figure 5:**
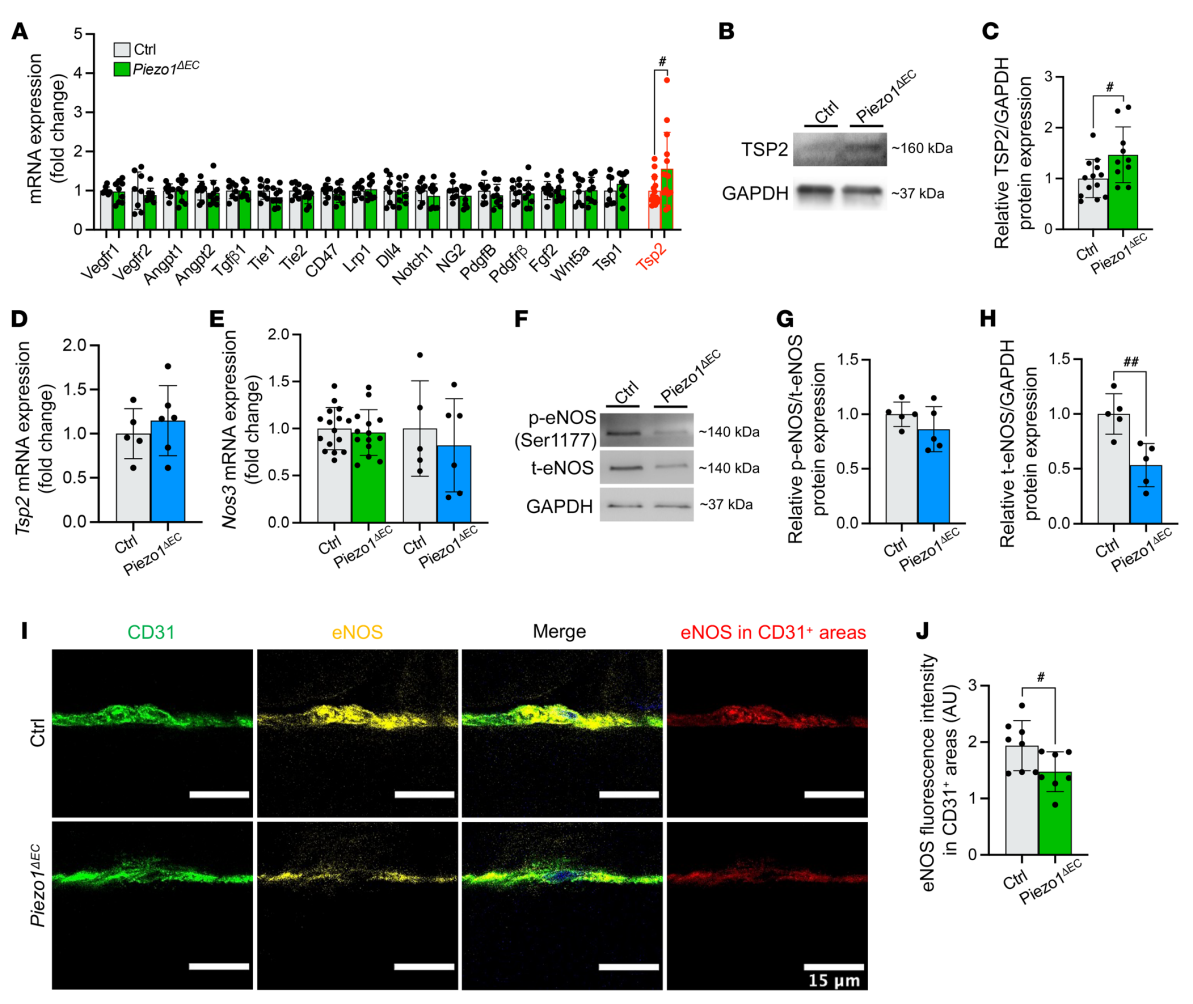
Downstream signaling mediated by eNOS and TSP2. (**A**) Relative mRNA levels for candidate downstream genes in whole gastrocnemius muscle from control (gray) and *Piezo1^ΔEC^* (green) mice. (**B**) Representative Western blot for TSP2 protein in gastrocnemius muscle. (**C**) For data of the type shown in **B**, quantification of TSP2 protein normalized to GAPDH and expressed as fold-change in *Piezo1^ΔEC^* compared to Ctrl. (**D **and** E**) Relative mRNA levels for (**D**) the *Tsp2* gene in isolated endothelial cells from skeletal muscle (SkECs) of Ctrl (gray) and *Piezo1^ΔEC^* (blue) mice and (**E**) eNOS (*Nos3* gene) mRNA in whole gastrocnemius muscle from Ctrl (gray) and *Piezo1^ΔEC^* (green) mice and isolated SkECs of Ctrl (gray) and *Piezo1^ΔEC^* (blue) mice. mRNA abundance was determined by qRT-PCR, normalized to housekeeping gene expression, and is presented as the fold-change relative to Ctrl mice. (**F**) Representative Western blot for eNOS phosphorylation at serine 1177 (p-eNOS) and total eNOS (t-eNOS) in isolated SkECs. (**G**) For data of the type shown in **F**, quantification of p-eNOS relative to t-eNOS in *Piezo1^ΔEC^* compared to Ctrl mice. (**H**) For data of the type shown in **F**, quantification of t-eNOS relative to the housekeeper protein GAPDH in *Piezo1^ΔEC^* compared to Ctrl. (**I**) Immunohistochemistry for CD31 (green) and eNOS (yellow) in gastrocnemius muscle longitudinal sections. Merged images are shown on the right. eNOS fluorescence intensity was measured in CD31^+^ regions (red). Scale bars: 15 μm. (**J**) Quantification of eNOS fluorescence intensity in CD31^+^ regions corresponding to endothelial cells. Data are for *n =* 8 to 9 mice per group (**A **and** F**–**H**), *n =* 10 to 13 (**B **and** C**), *n =* 5 to 6 (**D **and** E**), and *n =* 7 to 8 (**I **and** J**) (mean ± SD). Superimposed dots are the underlying data values for each mouse. ^#^*P* < 0.05, ^##^*P* < 0.01 vs. Ctrl mice. Statistical significance was evaluated using Student’s *t* test.

To identify signaling pathways mediating the apoptosis, we took whole muscle and quantified expression of 18 candidate genes that have been previously associated with exercise and vascular structure (Figure 5A). There was only one difference: an increase in thrombospondin-2 (*Tsp2*) mRNA (Figure 5A). There was a similar increase in TSP2 protein (Figure 5, B and C). TSP2 is a known inducer of apoptosis in microvascular endothelial cells (25, 26). TSP1 is similarly proapoptotic (26), but *Tsp1* expression was not modified by Piezo1 deletion (Figure 5A). We considered, therefore, that Piezo1 might be a selective negative regulator of *Tsp2* in endothelial cells. However, in SkECs the expression of *Tsp2* mRNA was unaffected by Piezo1 deletion (Figure 5D). Moreover, TSP2 protein could not be reliably detected in SkECs (Supplemental Figure 7A), indicating weak or no expression in this cell type. The data suggest that upregulated TSP2 is a mediator of the endothelial cell apoptosis in *Piezo1^ΔEC^* mice but not an important factor of endothelial cells or mediator of Piezo1’s effects in endothelial cells.

### Downregulation of endothelial nitric oxide synthase.

We hypothesized that endothelial nitric oxide (NO) synthase (eNOS) is the factor regulated by Piezo1 in SkECs because eNOS phosphorylation at a key serine residue is positively impacted by Piezo1 in arteries to mediate relaxation (18, 27, 28). NO is readily diffusible (29) and so could potentially act as a paracrine mediator to modulate TSP2 in adjacent cell types (30). It was previously shown that NO negatively regulates *Tsp2* gene expression in NIH3T3 fibroblasts (30). We found that the expression of the gene encoding eNOS, *Nos3*, was unaffected by *Piezo1^ΔEC^* in whole muscle or SkECs (Figure 5E). To our surprise, analysis of SkECs revealed no effect of *Piezo1^ΔEC^* on the amount of serine-phosphorylated eNOS relative to total eNOS (Figure 5, F and G). However, total eNOS protein was reduced (Figure 5, F and H). Furthermore, muscle longitudinal sections were stained for eNOS in addition to CD31 (Figure 5I), revealing that eNOS intensity was also decreased in situ (Figure 5J). The data suggest that Piezo1 in muscle endothelial cells is required to maintain the abundance of eNOS protein, which could account for the endothelial cell apoptosis in *Piezo1^ΔEC^* mice because eNOS produces NO and NO suppresses apoptosis (31), i.e., loss of eNOS could lead to loss of NO and therefore more apoptosis.

### Selective Tsp2 gene regulation by NO in muscle pericytes.

**Figure 6:**
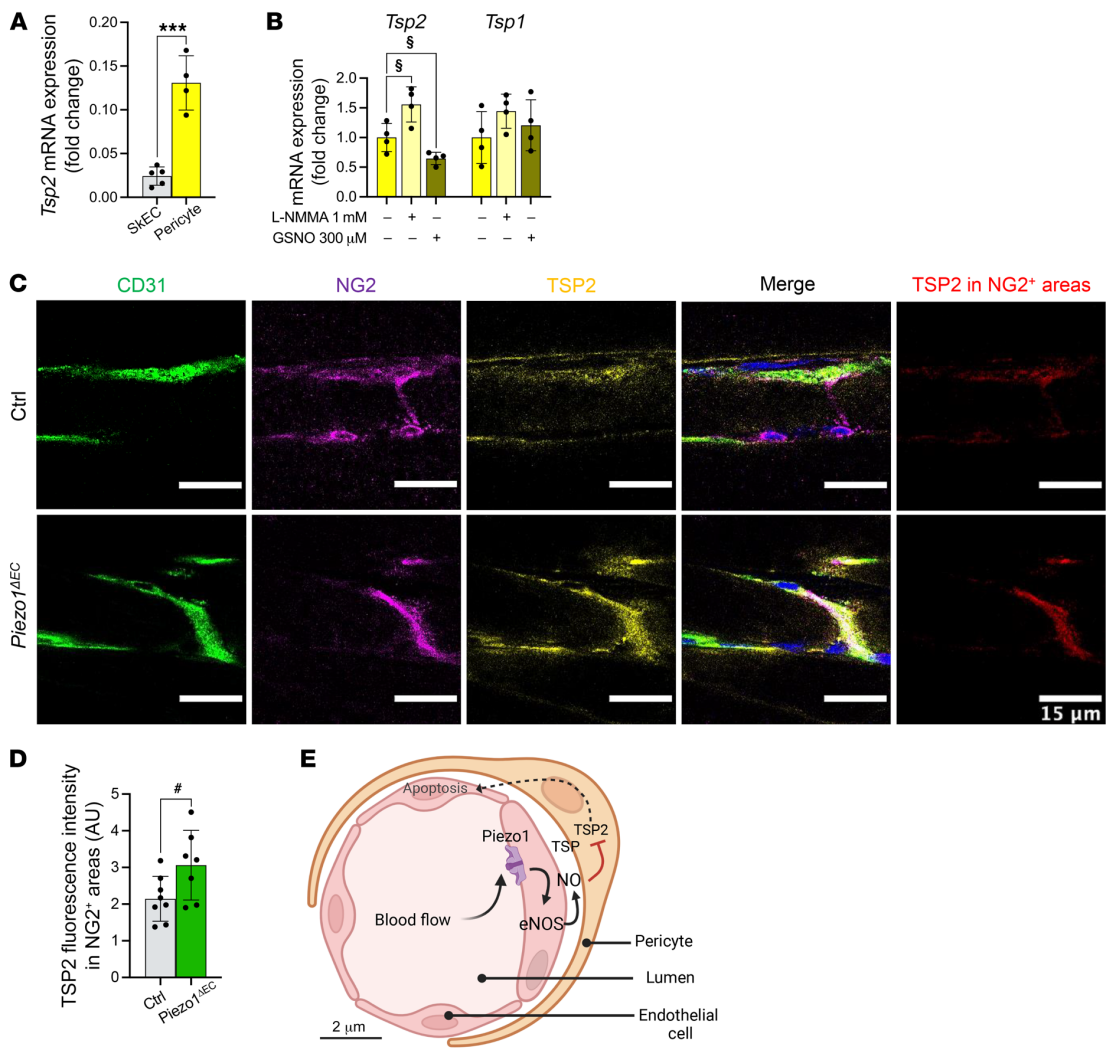
In situ upregulation of TSP2 in pericytes. (**A **and** B**) Relative mRNA abundance for (**A**) *Tsp2* in isolated SkECs (gray) and pericytes (yellow) from WT mice, and (**B**) *Tsp2* and *Tsp1* in isolated pericytes from WT mice: untreated (yellow), treated with NO inhibitor (1 mM L-NMMA, light yellow), or with NO donor (300 μM GSNO, dark yellow/green) for 4 hours. mRNA abundance was determined by qRT-PCR, normalized to housekeeping gene expression, and is presented as fold-change relative to isolated SkECs (**A**) or untreated pericytes (**B**). (**C**) Immunohistochemistry for CD31 (green), NG2 (magenta), and TSP2 (yellow) in gastrocnemius muscle longitudinal sections. Merged images are shown on the right. TSP2 fluorescence intensity was measured in NG2^+^ regions (red). Scale bars: 15 μm. (**D**) Quantification of TSP2 fluorescence intensity in NG2^+^ regions corresponding to pericytes. (**E**) Schematic model of the mechanism. Data are for *n =* 4 mice per group (**A** and **B**) and *n =* 7 to 8 (**C **and** D**) (mean ± SD). Superimposed dots are the underlying data values for each mouse. ^#^*P* < 0.05 vs. Ctrl mice; ***P < 0.001 vs. WT isolated SkECs; ^§^*P* < 0.05 vs. untreated WT isolated pericytes. Statistical significance was evaluated using Student’s *t* test.

Pericytes envelop muscle microvascular endothelial cells, and so we hypothesized that NO produced in endothelial cells might diffuse to regulate pericyte *Tsp2*. We therefore isolated and cultured pericytes from muscle (Supplemental Figure 7D). *Tsp2* mRNA abundance was 5 times greater in muscle pericytes compared with SkECs (Figure 6A) and TSP2 protein was readily detected (Supplemental Figure 7, B and C), making pericytes a candidate for the source of TSP2. Moreover, the pericytes showed increased *Tsp2* mRNA expression when basal NO production was suppressed by an L-arginine analog (NG-monomethyl-L-arginine, L-NMMA) and decreased *Tsp2* mRNA when excess NO was introduced by a NO donor, *S*-nitrosoglutathione (GSNO) (Figure 6B). There were no effects on *Tsp1* mRNA expression (Figure 6B). Muscle longitudinal sections were stained for TSP2, revealing that in situ pericyte–associated TSP2 was increased in *Piezo1^ΔEC^* mice (Figure 6, C and D). The data suggest pericytes as the source of TSP2-specific regulation by Piezo1, mediated by NO.

## Discussion

The results suggest that the longer (10-week) disruption of endothelial Piezo1 causes sustained depression of physical capability without altering desire for exercise or changing respiration, energy metabolism, cardiac function, or skeletal muscle mass or fiber type. This phenotype can be explained by capillary rarefaction in muscle. In our model (Figure 6E), shear stress from blood flow stimulates endothelial Piezo1, promoting eNOS stability in endothelial cells to keep the local NO concentration high. We suggest that NO then diffuses to adjacent pericytes where it suppresses *Tsp2* gene expression and thereby TSP2 and its proapoptotic effects (25, 26). TSP2 also has anti-eNOS effects (26), so loss of TSP2 would be expected to further elevate the local NO concentration. In the model, physical exercise increases blood flow, shear stress on the endothelium, activation of Piezo1 and therefore activation of eNOS/TSP2 paracrine signaling to increase the stability of microvascular endothelium and preserve, and potentially expand, microvascular architecture and muscle perfusion — increasing muscle performance. Loss of endothelial Piezo1 downregulates the axis, leading to loss of its stabilizing effects. Future studies could identify additional components of this axis and reveal other mechanisms that work in parallel or synergize with it. We do not exclude NO regulation of TSP2 expression in fibroblasts (30) but consider that the close proximity of pericytes to endothelial cells is likely to create a more efficient and potentially important local system for capillary regulation. We show that eNOS is the type of NOS regulated by Piezo1 in SkECs but we do not eliminate the possibility that other NOS types (32) could regulate and impact the TSP2 expression, for example in pericytes.

Consistent with our proposed mechanism, eNOS-knockout (eNOS-KO) mice show impaired physical performance (33, 34) as well as vessel rarefaction (35, 36) and reduced angiogenesis and arteriogenesis (37–39). eNOS is considered to have a key role in maintaining vascular integrity with aging (40). Apoptotic nuclei are rare in normal adult mouse skeletal tissue, but apoptosis rate increases with aging, such that endothelial cells account for more than 75% of the apoptotic cells in 25-month-old mice (41). eNOS-KO endothelial cells display an enhanced apoptosis induction, suggesting that eNOS downregulation may be involved in age-dependent increases in apoptosis sensitivity (42).

eNOS is dynamically regulated at multiple levels, although relatively little is known about mechanisms regulating the protein stability (43, 44). Our suggestion of an effect via regulation of eNOS protein stability is perhaps surprising but, in general, regulated degradation is crucial for controlling the abundance of many proteins and is mediated by an array of protein modification, sorting, and degradation enzymes (45). A key aspect is the ubiquitin-proteasome system, which has been linked to eNOS degradation in bovine endothelial cells (46). Another pathway involving protein kinase C, zeta type/extracellular signal–regulated kinase 5 (PKCζ/ERK5) was suggested to contribute to eNOS protein stability in human endothelial cells (47). How eNOS stability is regulated in muscle microvascular endothelial cells will be a future question of interest.

Our data show selectivity of endothelial Piezo1 for regulation of *Tsp2* with no effect on expression of *Tsp1* or other relevant genes tested. This might seem surprising when TSP1 is implicated in the control of skeletal muscle capillarity (48) and NO has wide-ranging effects (29). However, the *Tsp1* and *Tsp2* genes are regulated differently (49) and their disruption has opposite effects on wound healing, which is delayed in TSP1 KOs and accelerated in TSP2 KOs, suggesting that TSP1 can promote rather than inhibit angiogenesis under some conditions (50–52). TSP2 is established as a proapoptotic factor that inhibits vessel growth and stability (25, 26, 30, 51–55) and there is a compelling case for NO-dependent suppression of *Tsp2* gene expression (30), which is in keeping with the delayed wound healing after eNOS KO (37) and the opposite of TSP2 KO (52). This is consistent with the suggested reciprocal relationship between Piezo1 and TSP2, i.e., more Piezo1 expression or Piezo1 activity leading to more eNOS/NO, less TSP2, less endothelial cell apoptosis, and more capillarity; and conversely, less Piezo1 expression or activity leading to less eNOS/NO, more TSP2, more apoptosis and attrition of capillaries (i.e., rarefaction). Contributions from other, TSP2-independent mechanisms cannot be excluded. Our pericyte data suggest that the *Tsp2* gene in these cells is selectively regulated by NO, without effect on *Tsp1* gene expression, leading to the conclusion that endothelial Piezo1 achieves selective regulation of TSP2 in muscle microvasculature through selective NO regulation of the *Tsp2* gene in pericytes.

In this triad hypothesis of ours (Figure 6E), we suggest that Piezo1 is a key mechanical sensor of a blood flow, such that endothelial Piezo1 disruption prevents or compromises blood flow sensing. We suggest that a comparable situation arises when Piezo1 is normally expressed but muscle inactivity reduces its activation due to reduced blood flow. When an individual attempts to return to presedentary activity, microvascular perfusion and performance will be less than previously. Through retraining, and therefore renewed Piezo1 activation, the individual can gradually regain the original performance. Such a mechanism may exist to match muscle perfusion to usage, but it would come at a cost of reduced performance during prolonged inactivity, which might be unexpectedly needed later. It may be for this reason that the adaptation is relatively slow to occur, showing little impact after 2 weeks (19) but major impact after 10 weeks (this study). We think this long-term adaptive mechanism could be linked to decreased eNOS in endothelial cells. In an ischemia-induced angiogenesis model, the complete lack of eNOS in eNOS-KO mice started to affect the capillary density of thigh muscles after 2 weeks (56), so only a partial reduction in eNOS expression in quiescent cells as seen in our model could explain the slow establishment of capillary rarefaction.

Recently, random microvascular occlusion studies revealed that skeletal muscle is sensitive to reductions of greater than 10% in capillary flow, suggesting only a small functional reserve (23). Reductions greater than this led to steep decline in muscle performance (23). In our studies, endothelial Piezo1 deletion led to an approximately 20% reduction in capillary density, which is therefore consistent with rarefaction being the explanation for the observed poorer physical performance. We suggest that this concept is vascular bed specific because the cardiac capillary density was not affected by the endothelial Piezo1 deletion. This vascular bed specificity is consistent with prior eNOS studies showing that eNOS-KO mice showed no changes in basal coronary flow (57) and have normal cardiac hemodynamics (58).

Lower physical activity and performance are generally equated with increased disease risk and this is why, for example, contemporary societies emphasize the number of steps individuals perform per day (2). We speculate that endothelial Piezo1 is central to this biology because of the well-established importance of the endothelium and microvasculature in cardiovascular health (59, 60). An interpretation of our data is that we have effectively induced a type of genetic detraining despite the desire for continued exercise. Similarly, in humans there could be natural variability in the expression or functionality of Piezo1 — also what is effectively a type of genetic detraining. Such effects, if they exist, may contribute to why some people struggle to achieve or maintain physical performance. This is consistent with the idea that microvascular density, and thus quality of perfusion in muscle and other organs, is critical in cardiovascular health, metabolism and, importantly, quality of life generally (60, 61). There is now substantial evidence that capillary regression is a feature of aging humans and that it can be protected against by regular physical exercise training, resulting in slower decline in physical incapacity and onset of age-related diseases (61). Reduction in muscle microvascular density has been suggested to precede other muscle changes in heart failure patients and be critical in their exercise intolerance (62).

Piezo1 is widely expressed and known to have other, nonvascular functions (17) such as determining bone strength (63), cartilage force sensing (64), and myotube formation (65). Detection of whole-body exercise capability may therefore have a role in the link between weight-bearing exercise and the known benefits for protection against osteoporotic bone loss and cardiovascular disease. Moreover, Piezo1 is important in immunity and resistance to lung infection where its cyclical activation by pressure changes is suggested to be important (66). It may therefore facilitate exercise-related enhancement of protection against microbes. Overall, we speculate that Piezo1 senses physical vibrancy and couples it to health.

In conclusion, we suggest that endothelial Piezo1 is a critical molecule in maintaining muscle capillarity and therefore physical capability. In the future it will be useful to expand knowledge of how the sensitivity of Piezo1 is set and regulated in various contexts, including in aging, features of which are microvascular apoptosis (67) and exercise intolerance in common diseases of old age such as heart failure (68). It may become apparent how to achieve suitable intervention because pharmacological agonists of Piezo1 have already been identified, demonstrating the potential for enhancing sensitivity of the channels to mechanical force (27, 69, 70). The chemical properties of these tools currently restrict their general utility but refinement efforts and discovery of new modulators might lead to agents that protect against the adverse consequences of physical inactivity.

## Methods

### Piezo1-transgenic mice

*Piezo1^ΔEC^* mice and control littermates were housed in GM500 individually ventilated cages (Animal Care Systems) at 21°C, 50% to 70% humidity, and a 12-hour light/12-hour dark cycle on standard chow diet and water ad libitum. Genotypes were determined using real-time PCR with specific probes designed for each gene (Transnetyx Inc.). *Piezo1* conditional KO mice were generated at the University of Leeds by breeding C57BL/6J *Piezo1*-floxed mice (*Piezo1^fl/fl^*) with a Cre transgenic line driven by the cadherin-5 promoter (Tg[*Cdh5*-Cre/ERT2]1Rha) as previously described (18, 19). *Piezo1^fl/fl^* mice were crossed with *Cdh5*-Cre mice and inbred to generate Cre-positive, *loxP*-homozygous (*Piezo1^fl/fl^* *Cdh5*-Cre) conditional KO mice. Intraperitoneal injection of tamoxifen at 75 mg/kg for 5 consecutive days in *Piezo1^fl/fl^* *Cdh5*-Cre mice resulted in the disruption of the *Piezo1* gene specifically in the endothelium. These mice are referred to as *Piezo1^ΔEC^*. Control mice were the Cre-negative (*Piezo1^fl/fl^*) littermates that received tamoxifen injections. For experiments, male mice aged 10 weeks were injected with tamoxifen to induce the deletion of the *Piezo1* gene and studies were performed 10 weeks later, at which time mice were 20 weeks old.

### Echocardiography

Transthoracic echocardiography was performed using an echocardiograph (Vevo 2100 high-resolution system, VisualSonics) equipped with a 40-MHz linear MS-550D transducer, under steady-state isoflurane gas anaesthesia in 0.8 L/min 100% O_2_. The thickness of the left ventricular (LV) anterior and posterior walls was measured in the short axis using 2-dimensional-guided (2D) M-mode echocardiography over the entire cardiac cycle. The LV volumes, fractional shortening (FS %), ejection fraction (EF %), and the corrected LV mass were calculated with the Vevo LAB cardiac package software using the following equations: LV vol diastole (d) = (7.0/[2.4 + LVIDd]) × LVIDd^3^, LV vol systole (s) = (7.0/[2.4 + LVIDs]) × LVIDs^3^, FS % = 100 × ([LVIDd – LVIDs]/LVIDd), EF % = 100 × ([LV vol d – LV vol s]/LV vol d), and corrected LV mass = 0.8 × 1.053 × ([LVIDd + LVPWd + IVSd]^3^ – LVIDd^3^), where ID is the internal diameter, PW is posterior wall thickness, and IVS is the interventricular septum thickness.

### Metabolism, locomotor behavior, and physical activity

The Comprehensive Laboratory Animal Monitoring System (CLAMS, Columbus Instruments) was used to measure energy expenditure, locomotor behavior, and voluntary physical activity. Mice were housed individually in CLAMS metabolic cages equipped with running wheels (94 mm diameter), with free access to food and water, and maintained on a 12-hour light (inactive)/12-hour dark (active) cycle. They were acclimated for the first 24 hours and then data on O_2_ consumption, CO_2_ production, respiratory exchange ratio, energy expenditure, food intake, locomotor activity, and wheel revolutions were recorded for a further 72 hours in 10-minute bins. A mouse was considered active when wheel revolutions were recorded for a 10-minute bin.

### Animals and tissue harvest

Animals were euthanized in accordance with the Schedule 1 Code of Practice, UK Animals Scientific Procedures Act 1986. Hind limb muscles and hearts were removed, cleaned, dissected free of fat, and cut in half transversally. For immunohistochemistry, the distal part was mounted in optimal cutting temperature compound (Tissue-Tek) and snap-frozen in liquid nitrogen–cooled isopentane. For molecular biology, the proximal part was directly snap-frozen in liquid nitrogen. Samples were stored at –80°C until use.

### Isolation of SkECs

Skeletal muscle tissue (~500 mg) from the hind limb of 20-week-old mice was used. Tissue was manually cleaned of fat and cut into small pieces in DMEM. To combine enzymatic digestion and mechanical dissociation, a commercial murine skeletal muscle dissociation kit (Miltenyi Biotec, 130-098-305) was used in combination with the gentleMACS Octo Dissociator with heaters (Miltenyi Biotec, 130-096-427). Muscle pieces were transferred to C-tubes and digested with Miltenyi’s enzyme cocktail under agitation at 37°C for 61 minutes (program 37C_mr_SMDK_1). Samples were then filtered through a 70-μm strainer and washed with DMEM. To deplete CD45^+^ cells, the suspension was incubated for 15 minutes at 4°C under agitation with mouse CD45 microbeads (Miltenyi Biotec, 130-052-301) and then passed through an LS column (Miltenyi Biotec, 130-042-401). The cell suspension was then enriched for endothelial cells by incubating with mouse CD31 microbeads (Miltenyi Biotec, 130-097-418) for 15 minutes at 4°C under agitation and passed through an LS column. CD31^+^ cells retained in the LS column were eluted with PEB buffer (PBS, 2 mM EDTA, 0.5% BSA, pH 7.2) and centrifuged at 300*g* for 5 minutes. CD45^–^CD31^+^ cells corresponding to endothelial cells were directly pelleted for RNA or protein isolation (71–73).

### Isolation of pericytes from skeletal muscle

Skeletal muscle tissue (~500 mg) from the hind limb of 20-week-old C57BL/6J WT mice was used. Tissue was manually cleaned of fat and cut into small pieces in DMEM. Muscle pieces were digested with 1 mg/mL collagenase/Dispase in DMEM (Sigma-Aldrich, 10269638001) for 60 minutes under agitation at 37°C. Samples were then filtered through a 70-μm strainer and washed twice with growth medium MV2 (PromoCell, C-22022), supplemented with 10% fetal bovine serum and 1% penicillin-streptomycin solution. The cells were resuspended in MV2 medium and plated in gelatin-precoated flasks. Isolated cells were initially cultured in MV2 medium under conditions optimized for endothelial cells, but after 2 passages were switched to a medium optimized for pericyte growth (ScienCell, 1201). Pericytes were used after 1–2 additional passages. Pericytes were treated for 4 hours with 1 mM L-NMMA acetate (Tocris Bioscience, 0771) or 300 μM GSNO (Tocris Bioscience, 0603).

### Isolation of liver sinusoidal endothelial cells

Livers of 20-week-old C57BL/6J WT mice were used for preparation of liver sinusoidal endothelial cells (LSECs). Tissue was cut into small pieces and incubated under agitation at 37°C for 36 minutes with 0.1% collagenase II (Gibco, 17101-015) and Dispase solution (Gibco, 17105-041) using the gentleMACS Octo Dissociator with heaters (program 37C_mr_LIDK_1). Samples were then filtered successively through 100-μm and 40-μm strainers and washed twice with PEB buffer. The cell suspension was incubated with mouse CD146 microbeads (Miltenyi Biotec, 130-092-007) for 15 minutes at 4°C under agitation and passed through an LS column. CD146^+^ cells retained in the LS column were eluted with PEB buffer and centrifuged at 300*g* for 5 minutes. Cells were used after being cultured for 2 to 3 days in MV2 medium.

### RNA isolation and quantitative PCR

Total RNA from gastrocnemius muscle, isolated endothelial cells, or pericytes was extracted using TRIzol (Sigma-Aldrich, T9424) according to the manufacturer’s instructions. For whole muscle, cDNA was synthesized from 1 μg of total RNA and random hexamer primers (Promega, C1181), incubated at 75°C for 7 minutes to denature RNA, and cooled at room temperature for 10 minutes. A second mixture containing M-MLV Reverse Transcriptase Buffer (ThermoFisher Scientific, 18057018), deoxynucleotide triphosphates (ThermoFisher Scientific, R0192), RNase inhibitor (ThermoFisher Scientific, 10777019), and M-MLV Reverse Transcriptase (ThermoFisher Scientific, 28025013) was added to the first mixture and incubated for 1 hour at 37°C, followed by 5 minutes at 95°C to inactivate the enzyme reaction. For isolated endothelial cells and pericytes from skeletal muscle, 300 ng and 100 ng of total RNA was reverse transcribed, respectively, using the High-Capacity RNA-to-cDNA Kit (ThermoFisher Scientific, 4387406) according to the manufacturer’s instructions. cDNA was used as a template for real-time PCR with SYBR Green Supermix (Bio-Rad, 1725121). PCR cycling conditions were 95°C for 10 minutes, 40 cycles of 95°C for 10 seconds, and 60°C for 1 minute. Quantitative determination of mRNA expression levels was performed with a LightCycler 480 Real Time PCR System (Roche) using either gene-specific primers or *Rps20* gene primers for whole muscle and *Gapdh* gene primers for isolated cells as endogenous controls from Sigma-Aldrich (Supplemental Table 2). Samples were analyzed using the comparative CT method, where fold-change was calculated from the ΔΔCT values with the formula 2^–ΔΔCT^.

### Immunoblotting

Proteins from gastrocnemius muscle were isolated in RIPA buffer (50 mM Tris HCl pH 7.4, 150 mM NaCl, 1% Triton X-100, 0.05% NP-40, 1% deoxycholate, 0.1% SDS), and proteins from isolated endothelial cells and pericytes were isolated in NP40 Cell Lysis Buffer (ThermoFisher Scientific, FNN0021), both supplemented with protease inhibitor cocktail (Sigma-Aldrich, P8340) and phosphatase inhibitor cocktail Set V (Millipore, 524629). Samples were heated at 37°C for 30 minutes in SDS-PAGE sample buffer, loaded in a precast 4%–20% polyacrylamide gradient gel (Bio-Rad), and subjected to electrophoresis. Proteins were transferred onto a PVDF membrane for 90 minutes at 50 mA using the Trans-Blot SD Semi-Dry Electrophoretic Transfer Cell System (Bio-Rad). Membranes were blocked in 5% milk for 1 hour and then incubated overnight with the primary antibodies (Supplemental Table 3). Membranes were washed and incubated with secondary antibodies for 1 hour (Supplemental Table 3). Detection was performed using SuperSignal West Femto (ThermoFisher Scientific, 34096) and visualized with a G-Box Chemi-XT4 (SynGene). GAPDH was used as reference protein.

### Muscle processing and immunohistochemistry sectioning of skeletal muscle and heart muscle

Serial cross sections (10 μm thick) of gastrocnemius muscle and heart were cut in a cryostat (Leica) maintained at –20°C and mounted on SuperFrost Plus Adhesion slides (ThermoFisher Scientific, 10149870) to determine fiber type, fiber area, capillarization, pericyte density, vascular regression, and endothelial cell apoptosis. Longitudinal sections (30 μm thick) of gastrocnemius muscle were cut for eNOS and TSP2 immunohistochemistry experiments.

#### In situ determination of fiber type.

Muscle sections were immunolabeled for the different myosin heavy chains (MHCs) using a previously described method (74). Briefly, cross sections were either used to immunolabel for MHC type 1, 2a, and 2b or MHC type 2a and 2x. Sections were air dried at room temperature for 30 minutes, and then rehydrated with PBS. Sections were blocked using goat serum (10% in PBS) and incubated 2 hours at room temperature with one of the following primary antibody cocktails: (a) mouse IgG2b monoclonal anti–MHC type 1 (BA-F8), mouse IgG1 monoclonal anti–MHC type 2a (SC-71), and mouse IgM monoclonal anti–MHC type 2b (BF-F3); or (b) mouse IgG1 monoclonal anti–MHC type 2a (SC-71) and mouse IgM monoclonal anti–MHC type 2x (6H1). All primary antibodies targeting MHCs were purchased from the Developmental Studies Hybridoma Bank (DSHB, University of Iowa) and used at a concentration of 0.5 μg/mL. Muscle sections were washed 3 times in PBS and then incubated for 1 hour at room temperature with one of the following secondary antibody cocktails: (a) DyLight 405 IgG2b goat anti-mouse (Jackson Immunoresearch, 115-475-207; 1:500), Alexa Fluor 488 IgG1 goat anti-mouse (ThermoFisher Scientific, A-21121; 1:500), and Alexa Fluor 555 IgM goat anti-mouse (ThermoFisher Scientific, A-21426; 1:500); or (b) Alexa Fluor 488 IgG1 goat anti-mouse (1:500) and Alexa Fluor 555 IgM goat anti-mouse (1:500). Sections were washed 3 times in PBS and slides were coverslipped using ProLong Gold Antifade Mountant (ThermoFisher Scientific, P36934).

#### In situ determination of gastrocnemius muscle and heart capillarization and cross-sectional area.

Muscle sections were air dried for 30 minutes at room temperature and rehydrated with PBS prior to fixation with 4% paraformaldehyde (PFA) for 10 minutes. After washes with PBS, sections were permeabilized with 0.1% Triton X-100 for 10 minutes and then blocked with goat serum (5% in PBS) for 45 minutes. Sections were stained for 2 hours at room temperature with rat anti-CD31 (BD Biosciences, 550274; 1:100) or FITC-conjugated *Bandeiraea* *simplicifolia* isolectin B4 (IB4) (FITC-IB4, Sigma-Aldrich, L2895; 1:100) to detect blood vessels, in combination with rhodamine-conjugated WGA (Vector Laboratories, RL-1022; 1:100) to visualize the plasma membrane. Sections were washed 3 times in PBS and then incubated for 1 hour at room temperature with Alexa Fluor 647 chicken anti-rat (ThermoFisher Scientific, A-21472; 1:500). Sections were washed 3 times in PBS and slides were coverslipped using ProLong Gold Antifade Mountant.

#### In situ determination of vascular regression.

Muscle sections were air dried for 30 minutes at room temperature and rehydrated with PBS prior to fixation with 4% PFA for 10 minutes. After washes with PBS, sections were permeabilized with 0.1% Triton X-100 for 10 minutes and then blocked with BSA (5% in PBS) for 1 hour. First, sections were incubated for 1 hour at room temperature with rat anti-CD31 (1:100) and goat anti–Coll IV (Millipore, AB769; 1:50) for endothelial cells and basal lamina, respectively. Slides were washed 3 times in PBS and then incubated for 1 hour at room temperature with Alexa Fluor 647 chicken anti-rat (1:500) and FITC-conjugated donkey anti-goat (Jackson Immunoresearch, 705-095-147; 1:500). Sections were washed 3 times in PBS and slides were coverslipped using ProLong Gold Antifade Mountant.

#### In situ determination of pericyte density and coverage.

Muscle cross sections and longitudinal sections were air dried for 30 minutes at room temperature and rehydrated with PBS prior to fixation with 4% PFA for 10 minutes, and then permeabilized and blocked with PBS, 1% BSA, and 0.25% Triton X-100 for 1 hour at 4°C. Sections were incubated overnight at 4°C with rat anti-CD31 (1:100) and rabbit anti-NG2 proteoglycan (Millipore, AB5320; 1:250) for endothelial cells and pericytes, respectively. Slides were washed 3 times in PBS and then incubated for 1 hour at room temperature with Alexa Fluor 488 goat anti-rat (ThermoFisher Scientific, A-11006; 1:500) and Alexa Fluor 568 goat anti-rabbit (ThermoFisher Scientific, A-11011; 1:500). Sections were washed 3 times in PBS and slides were coverslipped using ProLong Gold Antifade Mountant.

#### In situ apoptosis determination.

Cellular apoptosis was detected on muscle sections using the in situ Cell Death Detection Kit, TMR Red (Sigma-Aldrich, 12156792910) as described by the manufacturer’s instructions. Then, muscle sections were counterstained for FITC-conjugated IB4 to identify endothelial cells. Muscle section slides were coverslipped using ProLong Gold Antifade Mountant with DAPI (ThermoFisher Scientific, P36935) to counterstain nuclei.

#### In situ determination of eNOS fluorescence intensity.

Longitudinal muscle sections were air dried for 30 minutes at room temperature and rehydrated with PBS prior to fixation with 4% PFA for 10 minutes, and then permeabilized and blocked with PBS, 1% BSA, and 0.25% Triton X-100 for 1 hour at 4°C. Sections were incubated overnight at 4°C with rat anti-CD31 (1:100) and mouse anti-eNOS (BD Biosciences, 610297; 1:100). Slides were washed 3 times in PBS and then incubated for 1 hour at room temperature with Alexa Fluor 488 goat anti-rat (1:500) and Alexa Fluor 647 goat anti-mouse (ThermoFisher Scientific, A-21235; 1:500). Sections were washed 3 times in PBS and slides were coverslipped using ProLong Gold Antifade Mountant with DAPI.

#### In situ determination of TSP2 fluorescence intensity.

Longitudinal muscle sections were air dried for 30 minutes at room temperature and rehydrated with PBS prior to fixation with 4% PFA for 10 minutes, and then permeabilized and blocked with PBS, 1% BSA, and 0.25% Triton X-100 for 1 hour at 4°C. Sections were incubated overnight at 4°C with rat anti-CD31 (1:100), rabbit anti-NG2 (1:250), and mouse anti-TSP2 (BD Biosciences, 611150; 1:100). Slides were washed 3 times in PBS and then incubated for 1 hour at room temperature with Alexa Fluor 488 goat anti-rat (1:500), Alexa Fluor 568 goat anti-rabbit (1:500), and Alexa Fluor 647 goat anti-mouse (1:500). Sections were washed 3 times in PBS and slides were coverslipped using ProLong Gold Antifade Mountant with DAPI.

#### In situ determination of pericyte and LSEC purity.

Cells were fixed with 4% PFA for 10 minutes. After washes with PBS, cells were permeabilized with 0.1% Triton X-100 for 5 minutes, and then blocked with 1% BSA in PBS for 15 minutes. Cells were stained for 1 hour at 37°C with rat anti–VE-cadherin (ThermoFisher Scientific, 14-1441-81; 1:200) and rabbit anti-NG2 (1:200). Cells were washed 3 times in PBS and then incubated for 30 minutes at room temperature with Alexa Fluor 647 goat anti-rabbit (ThermoFisher Scientific, A-21246; 1:200) and Alexa Fluor 568 goat anti-rat (ThermoFisher Scientific, A-11077; 1:200). Cells were washed and coverslipped using ProLong Gold Antifade Mountant with DAPI.

### Slide imaging and image quantification

For determination of fiber type, capillary density, pericyte density, cross-sectional area, vascular regression, and apoptosis, images were acquired using a confocal laser scanning microscope (Zeiss LSM 880) and ZEN (black edition) acquisition software, and then analyzed using ImageJ software version 1.52p (NIH). The percentage of fibers expressing the various MHC isoforms was manually calculated from 5 random fields containing an average of 200 fibers per field per animal (×20 magnification). Capillaries, pericytes, and fibers were counted in 10 random fields per animal (×20 magnification) using the 3D Objects Counter and Muscle Morphometry plugins, and capillary density was expressed as capillary-to-fiber ratio and pericyte density as pericyte-to-fiber ratio. The geometrical parameter, minimal Feret’s diameter, was used to determine muscle fiber size. Vascular regression was evaluated by the number of empty basement membrane sleeves (Coll IV^+^CD31^–^), as this process is characterized by extrusion of endothelial cells through the basal lamina, leaving only membrane sleeves without the blood vessel. Quantification was performed on 10 random fields per animal (×20 magnification). Apoptotic endothelial cells were detected by colocalized green (IB4^+^), red (TUNEL^+^), and blue (DAPI^+^) fluorescence. The percentage of apoptotic endothelial cells was calculated as IB4^+^TUNEL^+^DAPI^+^ cells in proportion to all IB4^+^DAPI^+^ cells. Quantification was performed on 15 random fields per animal (×40 magnification). For assessment of eNOS and TSP2 fluorescence intensity, imaging was carried out on a confocal laser scanning microscope (Zeiss LSM 710) using a 63×/1.40 NA oil objective. *Z*-slices of 1-Airy-unit thickness were used to obtain a 3D perspective of each vessel. Images were exported to Fiji (75) for final processing and assembly. For quantification of TSP2/eNOS intensities at the pericyte and endothelial regions, masks of each region were generated from the corresponding CD31 and NG2 images for each slice of the *Z*-stack. TSP2 intensity in pericytes was obtained by application of the NG2 mask. eNOS intensity in endothelial cells was obtained by application of the CD31 mask. NG2 and CD31 areas were calculated from the corresponding masks. Background subtraction on TSP2 images was carried out using the ImageJ background subtract function. For eNOS images, the intensity in unstained areas was measured and subtracted from the intensity values. TSP2 and eNOS fluorescence intensities were averaged from 2 to 10 *Z*-slice images per microvascular unit selected at random. Measurements from 2 to 9 microvascular units were then averaged to produce 1 fluorescence value per mouse. For assessment of pericyte and LSEC purity, imaging was carried out on a confocal laser scanning microscope (Zeiss LSM 710) using a 40×/1.3 NA oil objective. Images were exported and analyzed using ImageJ software. Pericytes were validated as NG2^+^VE-cadherin^–^ cells, while LSECs were used as VE-cadherin positive control.

### Data availability

All source data are provided.

### Statistics

The number (*n*) of mice studied per experiment is indicated in figure legends. All data are presented as mean ± SD. Outliers were removed in the validation analysis using the robust regression and outlier removal test (ROUT) method with *Q* = 1% in GraphPad Prism 9.0 software. Statistical significance was evaluated with unpaired Student’s *t* test when comparing 2 groups, and with 2-way ANOVA followed by post hoc Tukey’s test for multiple comparisons, as stated in figure legends. For metabolic studies, the statistical analysis was performed in the R programming language with CalR, a custom package for analysis of indirect calorimetry using ANCOVA (76). For all experiments, a *P* value of less than 0.05 was considered significant. For PCR analysis, each sample was tested in duplicate. Echocardiographic examinations were performed in a blinded fashion. In the CLAMS studies, 2 control mice were excluded from the analysis due to non-running behavior combined with weight loss of 10% or greater.

### Study approval

All animal use was authorized by the University of Leeds Animal Ethics Committee and The Home Office, UK.

## Author contributions

FB harvested the mice, performed the CLAMS experiments, muscle sectioning, immunohistochemistry on muscle and heart sections, mRNA extraction and qRT-PCR, SkEC, LSEC, and pericyte isolations, cell culture and treatments, analyzed CLAMS, echocardiography, qRT-PCR, and immunohistochemistry data, and cowrote the manuscript. MD harvested the mice, performed protein extraction, Western blotting, cell culture and treatments, and analyzed corresponding data. ECB performed immunocytochemistry on isolated cells and longitudinal sections and analyzed corresponding data. ELE performed echocardiography. KEM performed cell culture. GP provided technical assistance. PS, TSB, LCM, and MTK provided intellectual input. LL harvested the mice. TSF bred and maintained the mouse colonies. LDR raised research funds and assisted and advised on CLAMS experiments and analysis. DJB conceptualized the study, raised research funds, supervised the project team, and cowrote the manuscript.

## Supplementary Material

Supplemental data

## Version 1 01/13/2022

In-Press Preview

## Version 2 03/01/2022

Electronic publication
