# Supplementary material for: Endothelial Piezo1 sustains muscle capillary density and contributes to physical activity
Source: J Clin Invest. 2022 Mar 1;132(5):e141775. doi: 10.1172/JCI141775 (PMC8884896; doi:10.1172/JCI141775)
Supplement: Supplemental data [file jci-132-141775-s074.pdf]

Endothelial Piezo1 sustains muscle capillary density and contributes to physical activity

Fiona Bartoli[1], Marjolaine Debant[1†], Eulashini Chuntharpursat-Bon[1†], Elizabeth L Evans[1], Katie E Musialowski[1], Gregory Parsonage[1], Lara C Morley[1], T Simon Futers[1], Piruthivi Sukumar[1], T Scott Bowen[2], Mark T Kearney[1], Laeticia Lichtenstein[1], Lee D Roberts[1], David J Beech[1*]

[1]School of Medicine and [2]School of Biomedical Sciences, University of Leeds, Leeds, UK.

[†]Contributed equally.

*Author for correspondence: Professor David J Beech, Leeds Institute of Cardiovascular and Metabolic Medicine, School of Medicine, LIGHT Building, Clarendon Way, University of Leeds, Leeds LS2 9JT, England, UK. Telephone +44 113 343 4323. Email d.j.beech@leeds.ac.uk.

SUPPLEMENTAL INFORMATION (SI) – FIGURES

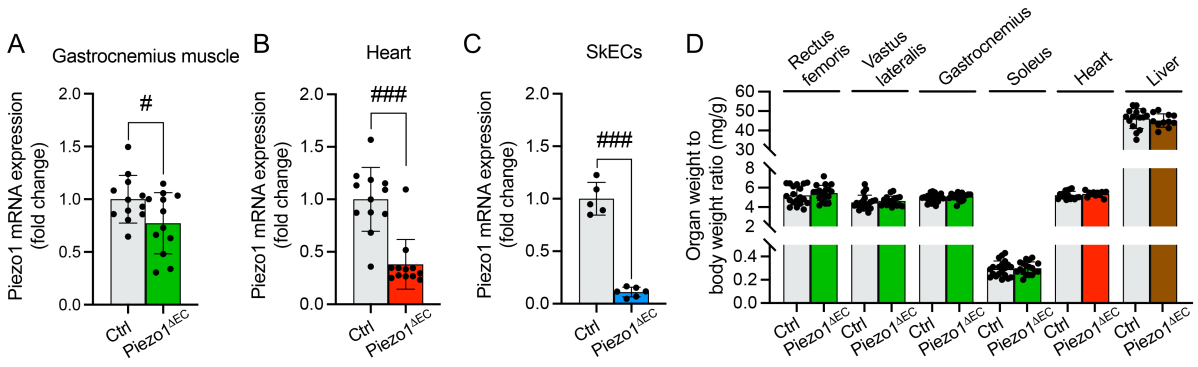

SI Figure 1: Anatomical parameters are normal in *Piezo1^{ΔEC}* mice despite efficient depletion of endothelial Piezo1. A-C, Quantitative PCR mRNA expression data for *Piezo1* gene in whole gastrocnemius muscle **(A)**, heart **(B)** and isolated endothelial cells from skeletal muscle (SkECs) **(C)** from Ctrl (grey) and *Piezo1^{ΔEC}* mice (colour). RNA abundance was normalized to housekeeping gene expression and presented as the fold-change relative to that in Ctrl mice. **D,** Organ weight to body weight ratio for: rectus femoris; vastus lateralis; gastrocnemius; soleus muscles; heart and liver from Ctrl (grey) and *Piezo1^{ΔEC}* mice (colour). Data are for **(A-B)** N = 12; **(C)** N = 5 – 6 and **(D)** N = 11 - 20 mice per group (mean ± S.D.). Superimposed dots are the individual underlying data values for each individual mouse. #P<0.05, ###P<0.001 *vs.* Ctrl mice. Statistical significance was evaluated using Student's *t*-test.

A

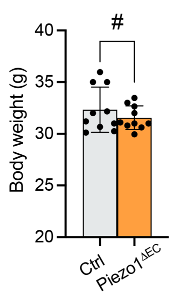

B

GLM

	Light			Dark		
Effect	Mass	Group	Interaction	Mass	Group	Interaction
Hourly Food Consumed (kcal)	0.0868	0.4376		0.5689	0.9615	
Total Food Consumed (kcal)	0.4281	0.8637		0.4243	0.9822	
Oxygen Consumption (ml/hr)	0.0297 *	0.1583		0.0360 *	0.1310	
Carbon Dioxide Production (ml/hr)	0.0780	0.2612		0.0954	0.2431	
Energy Expenditure (kcal/hr)	0.8021	0.2886		0.7705	0.2232	
Energy Balance (kcal/hr)	0.2865	0.5681		0.4729	0.6944	

ANOVA

	Light	Dark
Effect	Group	Group
Respiratory Exchange Ratio	0.8698	0.7065

Mass effect: subject.mass.x
Signif. Codes: <0.001 ***, <0.01 **, <0.05 *

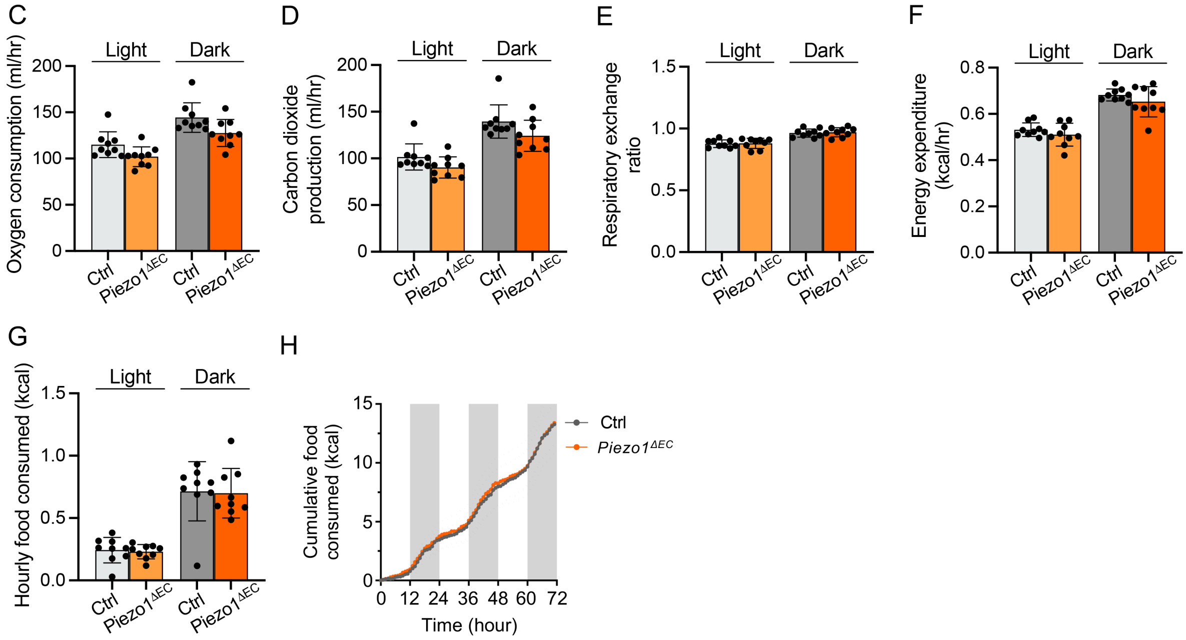

SI Figure 2: Metabolism and respiration are normal in *Piezo1$^{\Delta EC}$* mice. Throughout the figure, data in grey are for Ctrl mice and data in orange are for *Piezo1$^{\Delta EC}$* mice. Lighter colour is for data sampled during the light cycle and darker colour for data during the dark cycle. Data were measured for 3 light and dark cycles. **A,** Body weight data for mice used for CLAMS experiments. **B,** ANCOVA analysis of metabolism parameters using CalR software. **C,** Pooled, averaged, oxygen consumption data. **D,** Carbon dioxide production data. **E,** Respiratory exchange ratio data. **F,** Energy expenditure data. **G,** Food consumption data. **H,** Cumulative food consumption during 72 hr recording. Grey shaded areas indicate the dark cycles. Data are for N = 9 - 10 mice per group (mean ± S.D.). Superimposed dots are the individual underlying data values for each individual mouse. #*P*<0.05 *vs.* Ctrl mice. Statistical significance was evaluated using analysis of covariance (ANCOVA) with CalR software.

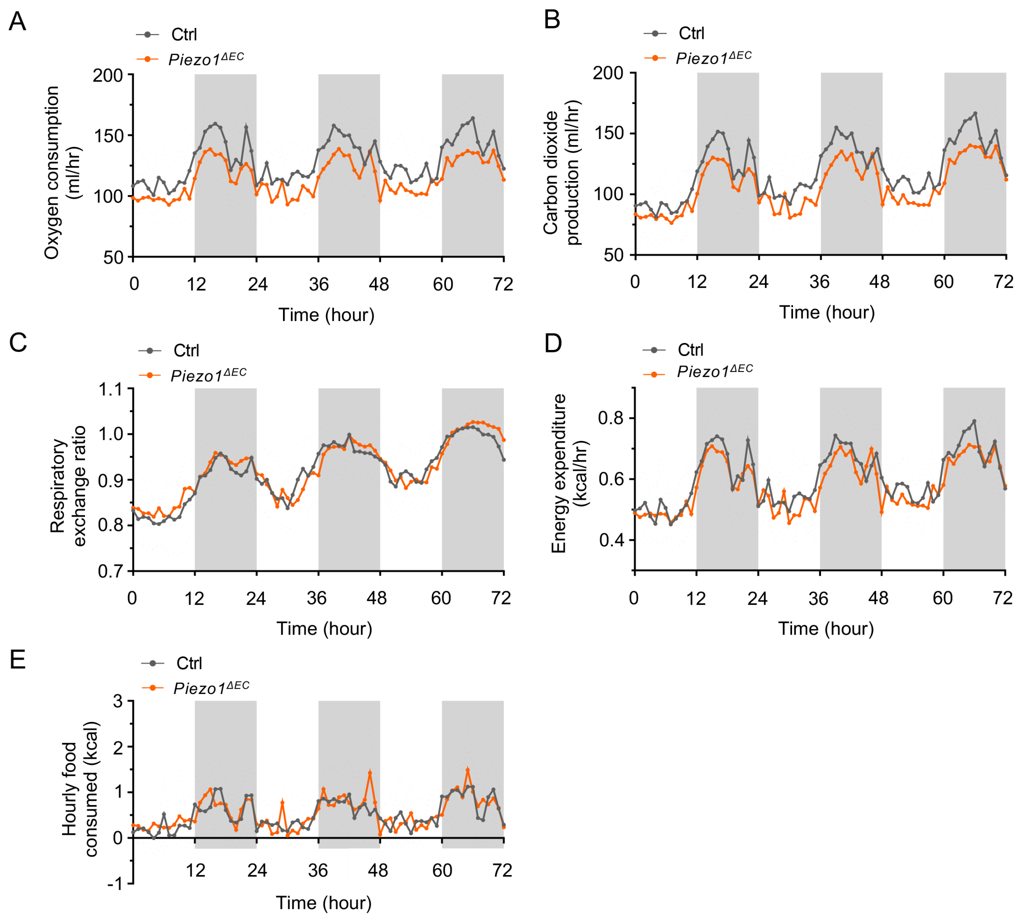

SI Figure 3: Circadian patterns of metabolic parameters are normal in *Piezo1$^{\Delta EC}$* mice. Throughout the figure, data in grey are for Ctrl mice and data in orange are for *Piezo1$^{\Delta EC}$* mice. Grey shaded areas indicate the dark cycles. **A,** Oxygen consumption data. **B,** Carbon dioxide production data. **C,** Respiratory exchange ratio data. **D,** Energy expenditure data. **E,** Food consumption data. Data are for N = 9 - 10 mice per group (mean ± S.D.).

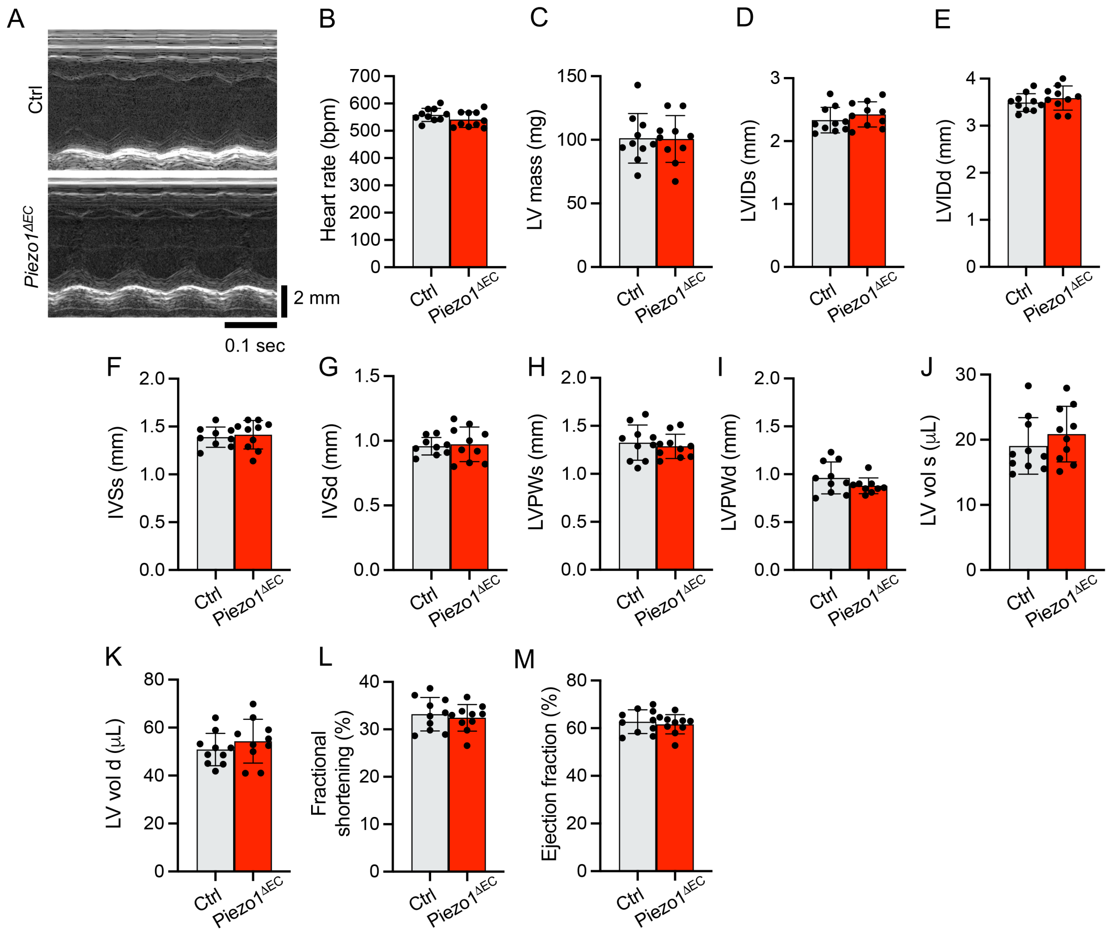

SI Figure 4: Normal cardiac structure and function in *Piezo1*$^{\Delta EC}$ mice. Data are for Ctrl mice (grey) and matched *Piezo1*$^{\Delta EC}$ mice (red). **A,** Representative echocardiography images of LV in short axis and in M-mode from Ctrl and *Piezo1*$^{\Delta EC}$ mice. Parameters obtained from analysis of echocardiograms from Ctrl and *Piezo1*$^{\Delta EC}$ mice. (**B**-**M**) Based on data of the type shown in (**A**): **B,** Heart rate; **C,** Corrected left ventricular (LV) mass; **D,** Left ventricular internal diameter in systole (LVIDs); **E,** LVID in diastole (LVIDd); **F,** Interventricular septum thickness at end-systole (IVSs); **G,** IVS in end-diastole (IVSd); **H,** Left ventricular posterior wall thickness in systole (LVPWs); **I,** LVPW in diastole (LVPWd); **J,** Left ventricular volume in systole (LV vol s); **K,** LV vol in diastole (LV vol d); **L,** Cardiac fractional shortening; **M,** Cardiac ejection fraction. Data are for N = 10 mice per group (mean ± S.D.). Superimposed dots are the individual underlying data values for each individual mouse.

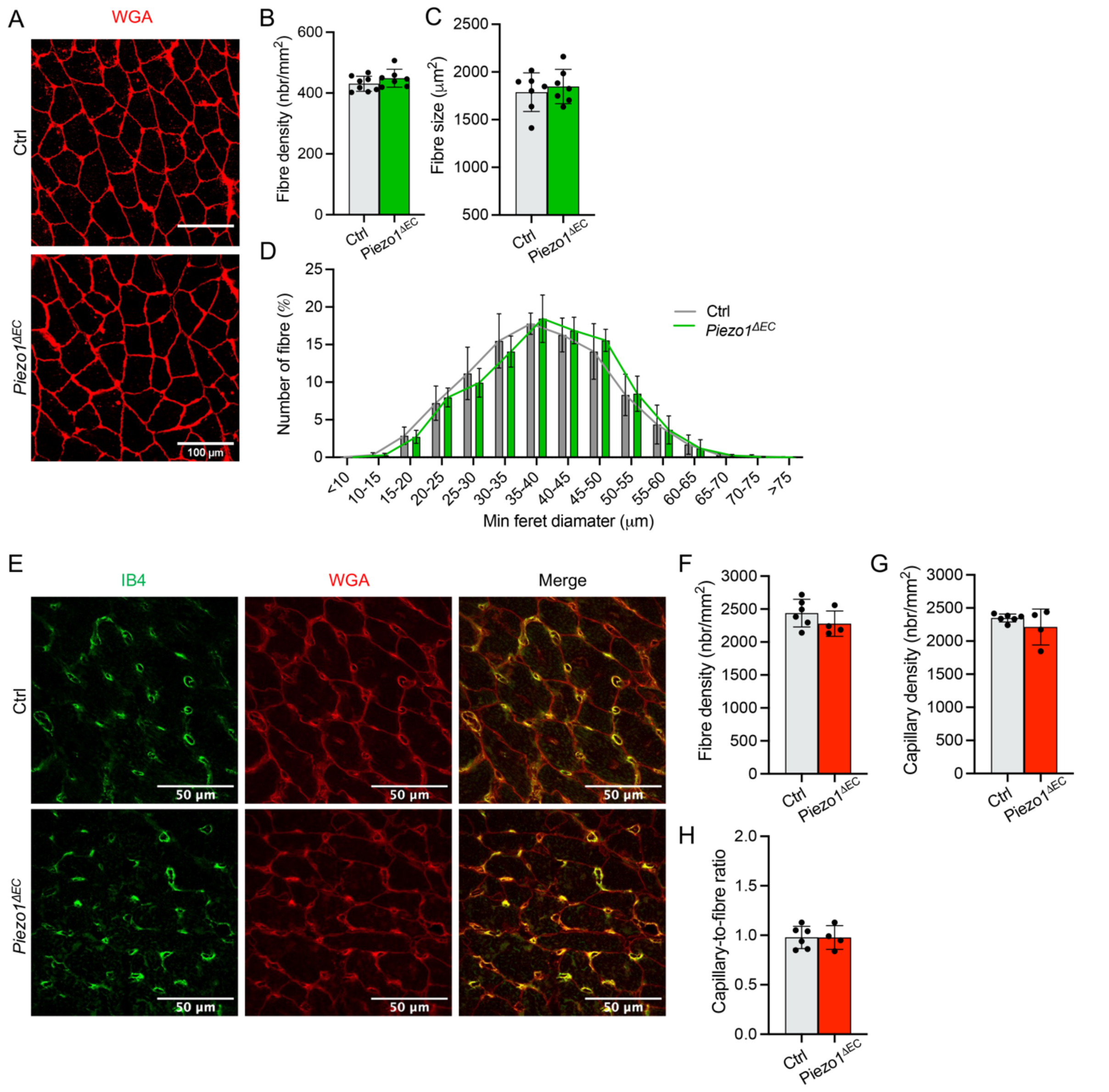

SI Figure 5: Normal skeletal fibre properties and heart capillary density in *Piezo1*$^{\Delta EC}$ mice. A, Immunohistochemistry for staining by wheat germ agglutinin (WGA, red) in gastrocnemius muscle sections of Ctrl and *Piezo1*$^{\Delta EC}$ mice. Scale bars, 100 µm. **B,** Fibre density. **C,** Fibre area. **D,** Fibre size distribution of gastrocnemius muscle from Ctrl (grey) and *Piezo1*$^{\Delta EC}$ mice (green), determined using the geometrical parameter minimum Feret's diameter. **E,** Immunohistochemistry for staining by isolectin B4 (IB4, green) and wheat germ agglutinin (WGA, red) in heart sections of Ctrl and *Piezo1*$^{\Delta EC}$ mice. Merged images are shown on the right. Scale bars, 50 µm. **F,** Fibre density. **G,** Capillary density. **H,** Capillary-to-fibre ratio measured from Ctrl (grey) and *Piezo1*$^{\Delta EC}$ mice (red) images of the type shown in (**E**). Data are for **(A-D)** N = 7 – 8 and (**E-H)** N = 4 – 6 mice per group (mean ± S.D.). Superimposed dots are the individual underlying data values for each individual mouse.

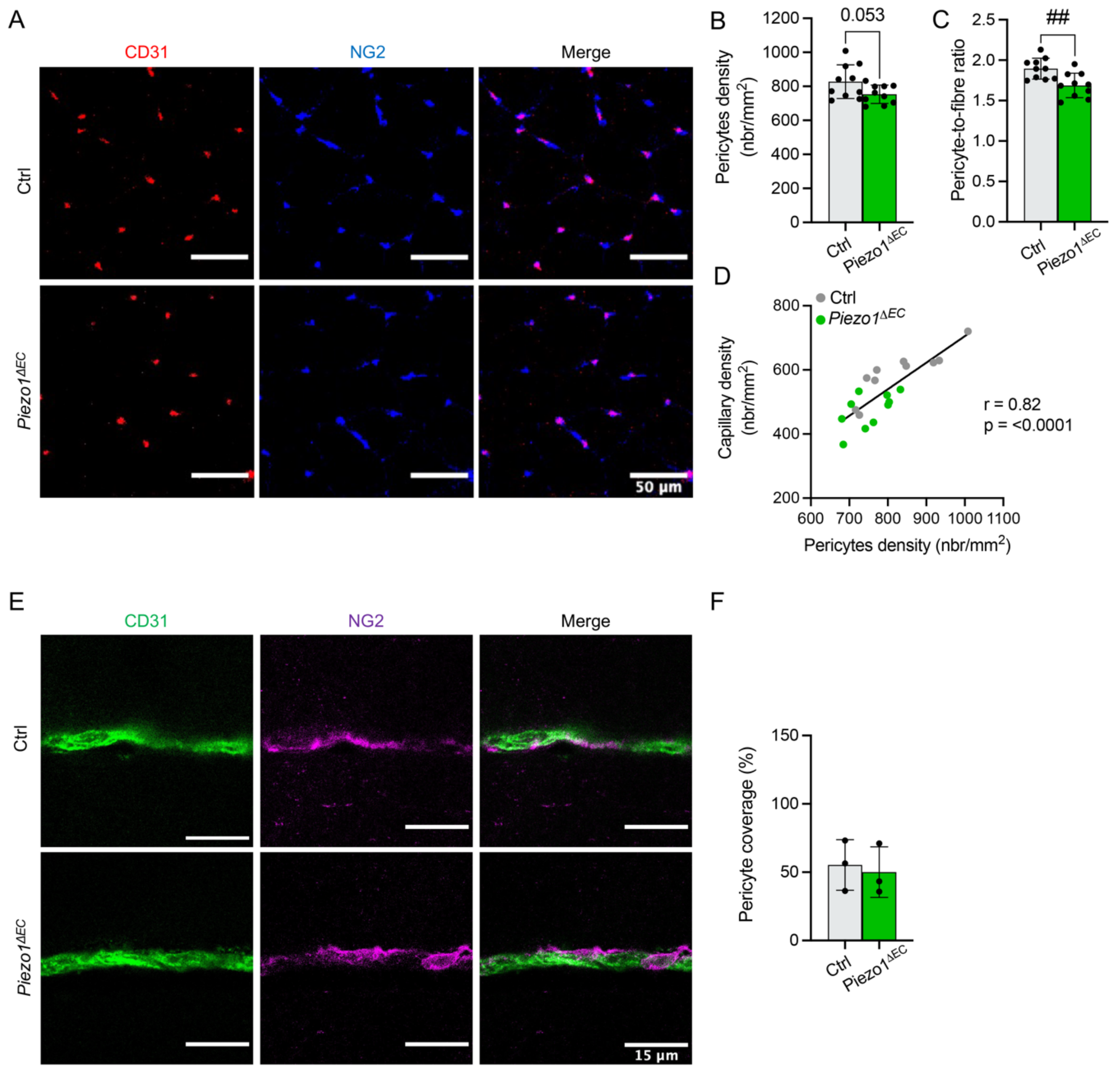

SI Figure 6: Decreased pericyte density but normal pericyte coverage of blood vessels in *Piezo1$^{\Delta EC}$* mice. A, Immunohistochemistry for staining by CD31 (endothelial cells, red) and NG2 (pericytes, blue) in gastrocnemius muscle cross-sections of Ctrl and *Piezo1$^{\Delta EC}$* mice. Merged images are shown on the right. Scale bars, 50 µm. **B,** Pericytes density. **C,** Pericyte-to-fibre ratio measured from Ctrl (grey) and *Piezo1$^{\Delta EC}$* mice (green) images of the type shown in (**A**). **D,** Pearson correlation of capillary and pericyte densities (r = 0.82, p < 0.0001). The black line is the correlation fit. **E,** Immunohistochemistry for staining by CD31 (endothelial cells, green) and NG2 (pericytes, magenta) in gastrocnemius muscle longitudinal sections of Ctrl and *Piezo1$^{\Delta EC}$* mice. Merged images are shown on the right. Scale bars, 15 µm. **F,** Quantification of pericyte coverage calculated as the percentage of NG2 positive cells in CD31 areas. Data are for (**A-D**) N = 10 and **(E-F)** N = 3 mice per group (mean ± S.D.). Superimposed dots are the underlying data values for each individual mouse. Grey: muscles from Ctrl mice. Green: muscles from *Piezo1$^{\Delta EC}$* mice. ##*P*<0.01 *vs.* Ctrl mice. Statistical significance was evaluated using Student's *t*-test except in **D** where Pearson's correlation was used.

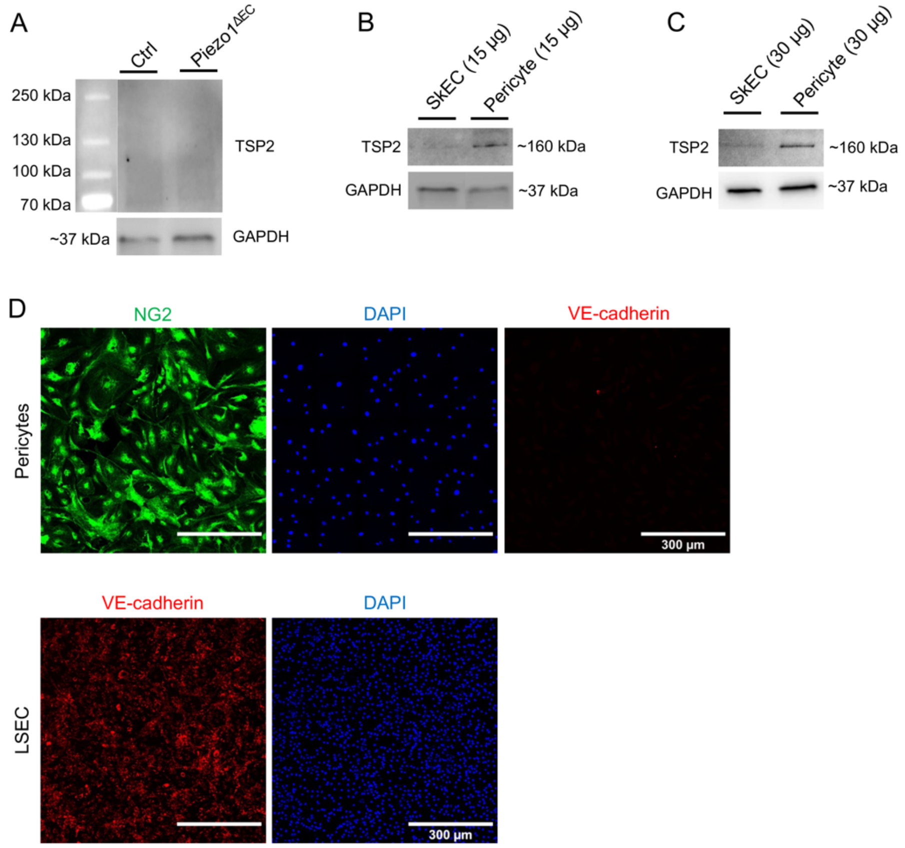

SI Figure 7: TSP2 expression in pericytes and pericyte culture purity. A, Representative western-blot for TSP2 protein in endothelial cells isolated from muscle (SkECs) of Ctrl and *Piezo1*$^{\Delta EC}$ mice. No specific band was detected. Data are for N = 3 mice per group. **(B-C)** Validation of the anti-TSP2 antibody used in (**A**) by western-blot in isolated pericytes from skeletal muscle compared to SkECs (**B**, 15 µg and **C,** 30 µg protein loaded). A specific band was detected in pericytes for TSP2 at approximately 160 kDa. Data are for N = 2 mice. **D,** Top: Immunohistochemistry for NG2 (green), DAPI (blue) and VE-cadherin (red), to visualize respectively pericytes, nuclei and endothelial cells in pericytes culture. Bottom: Immunohistochemistry for VE-cadherin (red) and DAPI (blue) in isolated endothelial cells from liver (LSECs) cultures, used as positive control for VE-cadherin antibody. Scale bars, 300 µm. Data are for N = 3 mice per condition.

SUPPLEMENTAL INFORMATION (SI) - TABLES

SI Table 1: Gene expression that was not different in *Piezo1*$^{\Delta EC}$ mice. The genes indicated were selected as markers of fibrosis, ER stress, fibre growth, fibre switch, hypoxia, inflammation, glucose and lipid metabolism, mitochondria biogenesis and immune cells. Gene expression was determined by RT-qPCR in whole gastrocnemius muscle from Ctrl and *Piezo1*$^{\Delta EC}$ mice. The abundance of mRNA was normalized to housekeeping genes and expressed as fold-change relative to controls. All displayed values are mean ± S.D. Statistical significance was evaluated using Student's *t*-test.

Gastrocnemius muscle						
Genes (common name)	Gene (official name)	Control mice	N	*Piezo1*$^{\Delta EC}$ mice	N	P value
Fibrosis						
Col1a1	*Col1a1*	1 ± 0.24	8	0.94 ± 0.29	9	0.65
Col3a1	*Col3a1*	1 ± 0.72	8	1.05 ± 0.46	9	0.87
Col4a1	*Col4a1*	1 ± 0.30	8	0.96 ± 0.26	9	0.79
Col4a2	*Col4a2*	1 ± 0.34	8	0.85 ± 0.21	9	0.29
ER stress						
Ire1	*Ern1*	1 ± 0.30	8	0.94 ± 0.33	9	0.70
Grp78	*Hspa5*	1 ± 0.17	8	0.91 ± 0.19	9	0.31
Atf6	*Atf6*	1 ± 0.17	8	0.84 ± 0.21	9	0.11
Fibre growth						
Icam1	*Icam1*	1 ± 0.30	8	0.92 ± 0.28	9	0.58
Murf1	*Trim63*	1 ± 0.36	8	0.97 ± 0.24	9	0.87
Atrogin1	*Fbxo32*	1 ± 0.43	8	0.97 ± 0.42	9	0.89
Fibre switch						
Myh1	*Myh1*	1 ± 0.46	8	1.02 ± 0.20	9	0.92
Myh2	*Myh2*	1 ± 0.16	8	0.91 ± 0.18	9	0.27
Myh4	*Myh4*	1 ± 0.38	8	1.19 ± 0.35	9	0.30
Myh7	*Myh7*	1 ± 0.21	8	0.81 ± 0.25	9	0.11
Pgc1α	*Ppargc1a*	1 ± 0.45	8	0.99 ± 0.44	9	0.95
Hypoxia / Inflammation						
Hif1α	*Hif1α*	1 ± 0.22	8	0.97 ± 0.26	9	0.81
Hif2α	*Epas1*	1 ± 0.27	8	1.03 ± 0.50	9	0.88
Glucose and lipid metabolism						
Glut4	*Slc2a4*	1 ± 0.42	8	0.83 ± 0.36	9	0.37
Pdk4	*Pdk4*	1 ± 0.31	8	1.24 ± 0.84	9	0.46
Pparα	*Pparα*	1 ± 0.38	8	1.01 ± 0.45	9	0.95
Pparγ	*Pparγ*	1 ± 0.41	8	1.19 ± 0.62	9	0.47
Cpt1b	*Cpt1b*	1 ± 0.21	8	0.89 ± 0.24	9	0.32
Cpt2	*Cpt2*	1 ± 0.38	8	0.78 ± 0.29	9	0.21
Cd36	*Cd36*	1 ± 0.25	8	1.01 ± 0.51	9	0.96
Hmgcr	*Hmgcr*	1 ± 0.24	8	0.94 ± 0.23	9	0.61
Fatp1	*Slc27a1*	1 ± 0.35	8	0.86 ± 0.37	9	0.44
Mitochondrial biogenesis						
Nrf1	*Nfe2l1*	1 ± 0.21	8	1.05 ± 0.33	9	0.70
Ucp3	*Ucp3*	1 ± 0.33	8	1.15 ± 0.58	9	0.53
Tfam	*Tfam*	1 ± 0.16	8	0.94 ± 0.15	9	0.47
Immune cells						
Cd11b	*Itgam*	1 ± 0.05	8	1.23 ± 0.15	9	0.19
Cd206	*Mrc1*	1 ± 0.08	8	1.16 ± 0.17	9	0.42
Cd45	*Ptprc*	1 ± 0.05	8	1.24 ± 0.19	9	0.28
F4/80	*Adgre1*	1 ± 0.10	8	1.19 ± 0.14	9	0.31

SI Table 2: qPCR primers.

Species	Gene (common name)	Gene (official name)	Forward (5'-3')	Reverse (5'-3')
Mus musculus	*Angpt1*	*Angpt1*	CATTCTTCGCTGCCATTCTG	GCACATTGCCCATGTTGAATC
	Angpt2	*Angpt2*	CCAACTCCAAGAGCTCGGTT	CGGTGTTGGATGACTGTCCA
	Atf6	*Atf6*	CGGTCCACAGACTCGTGTTC	GCTGTCGCCATATAAGGAAAGG
	Atrogin1	*Fbxo32*	CGTCTCACTTTCCCCTCAAG	GACTCCCAGCCATCCAATTAG
	Bak	*Bak1*	CCTTCGGGGTCTTCGTCTTT	ACCGTCACTTGTCACCTGAAT
	Bax	*Bax*	CAAACTGGTGCTCAAGGCCC	TCTTGGATCCAGACAAGCAGC
	Bcl2	*Bcl2*	TCTCAGTGAAGCCGGAGTGT	ACAACTTGCAATGAATCGGGAG
	BclXL	*Bcl2l1*	AACATCCCAGCTTCACATAACCCC	GCGACCCCAGTTTACTCCATCC
	Cd11b	*Itgam*	CAGCCCTAGCCTTGTGTCAT	GCTGCAACAACCACACTGG
	Cd206	*Mrc1*	TTCAGCTATTGGACGCGAGG	GAATCTGACACCCAGCGGAA
	Cd36	*Cd36*	GAGCAACTGGTGGATGGTTT	GCAGAATCAAGGGAGAGCAC
	Cd45	*Ptprc*	TGCAAGTGGAGGCACAGTA	GGTCACTGGGTGGATCTCTCT
	Cd47	*Cd47*	GGTGGGAAACTACACTTGCG	AGAAAACCACGAAACCGTGC
	Col1a1	*Col1a1*	GCTCCTCTTAGGGGCCACT	CCACGTCTCACCATTGGGG
	Col3a1	*Col3a1*	CTGTAACATGGAAACTGGGGAAA	CCATAGCTGAACTGAAAACCACC
	Col4a1	*Col4a1*	CTGGCACAAAAGGGACGAG	ACGTGGCCGAGAATTTCACC
	Col4a2	*Col4a2*	CCCGGATCTGTACAAGGGTG	CGCCTTTTGAGATTACGCCG
	Cpt1b	*Cpt1b*	GCTTAGTCGGGAGGCTCTGA	ACACCCCTAAGGATGCCATTC
	Cpt2	*Cpt2*	GGATAAACAGAATAAGCACACCA	GAAGGAACAAAGCGGATGAG
	Dll4	*Dll4*	GGAACCTTCTCACTCAACATCC	CTCGTCTGTTCGCCAAATCT
	F4/80	*Adgre1*	TGGCTGCCTCCCTGACTTTC	CAAGTGTACAGAAGGAAGCATAACC
	Fatp1	*Slc27a1*	CTGTAGCCAACCTGTTCCG	CTCCCCGCCATAAATGAGGG
	Fgf2	*Fgf2*	GGCTGCTGGCTTCTAAGTGT	TCTGTCCAGGTCCCGTTTTG
	Gapdh	*Gapdh*	TGAAGCAGGCATCTGAGGG	CGAAGGTGGAAGAGTGGGA
	Grp78	*Hspa5*	ACTTGGGGACCACCTATTCCT	ATCGCCAATCAGACGCTCC
	Glut4	*Slc2a4*	GGAAGGAAAAGGGCTATGCTG	TGAGGAACCGTCCAAGAATGA
	Hif1α	*Hif1α*	ACCTTCATCGGAAACTCCAAAG	CTGTTAGGCTGGGAAAAGTTAGG
	Hif2α	*Epas1*	TAAAGCGGCAGCTGGAGTAT	ACTGGGAGGCATAGCACTGT
	Hmgcr	*Hmgcr*	AGCTTGCCCGAATTGTATGTG	TCTGTTGTGAACCATGTGACTTC
	Icam1	*Icam1*	TGTGCTTTGAGAACTGTGGCA	TGGCGGCTCAGTATCTCCTC
	Ire1	*Ern1*	ACACTGCCTGAGACCTTGTTG	GGAGCCCGTCCTCTTGCTA
	Lrp1	*Lrp1*	ATGAGCTGGACGTGTGACAAGG	GCCTCTGAGCAGAACTTGTTGC
	Murf1	*Trim63*	AGTGTCCATGTCTGGAGGTCGTTT	ACTGGAGCACTCCTGCTTGTAGAT
	Myh1	*Myh1*	AATCAAAGGTCAAGGCCTACAA	GAATTTGGCCAGGTTGACAT
	Myh2	*Myh2*	AAGCGAAGAGTAAGGCTGTC	CTTGCAAAGGAACTTGGGCTC
	Myh4	*Myh4*	GAAGAGCCGAGAGGTTCACAC	CAGGACAGTGACAAAGAACGTC
	Myh7	*Myh7*	CTACAGGCCTGGGCTTACCT	TCTCCTTCTCAGACTTCCGC
	Ng2	*Cspg4*	CAGGCCTGCAAATCTGGGAG	GTCTTCTGGGCCCGAATCAT
	Nos3	*Nos3*	TCAGCCATCACAGTGTTCCC	ATAGCCCGCATAGCGTATCAG
	Notch1	*Notch1*	ATCAAGCGCTCTACAGTGGG	AGACAATGGAGCCACGGATG
	Nrf1	*Nfe2l1*	GCACCTTTGGAGAATGTGGT	CTGAGCCTGGGTCATTTTGT
	Pdgfrβ	*Pdgfrβ*	GCAGAAGAAGCCACGCTATG	CAGGTGGAGTCGTAAGGCAA
	Pdk4	*Pdk4*	GAGGATTACTGACCGCCTCTTTAG	TTCCGGGAATTGTCCATCAC
	Pgc1α	*Ppargc1a*	AGCCGTGACCACTGACAACGAG	GCTGCATGGTTCTGAGTGCTAAG
	Pparα	*Pparα*	TATTCGGCTGAAGCTGGTGTAC	CTGGCATTTGTTCCGGTTCT
	Pparγ	*Pparγ*	CACAATGCCATCAGGTTTGG	GCTGGTCGATATCACTGGAGATC
	Rps20	*Rps20*	GGACTTGATCAGAGGCGCCAAGGAAA	CCCAGGTCTTGGAACCTTCACCACAA
	Tfam	*Tfam*	CAAGTCAGCTGATGGGTATGG	TTTCCCTGAGCCGAATCATCC
	Tgfβ1	*Tgfβ1*	CCCTATATTTGGAGCCTGGA	CTTGCGACCCACGTAGTAGA
	Tie1	*Tie1*	GTAGGTTCCGTCTCTGCCAC	GGTCTCTTCACCCGATCCTG
	Tie2	*Tek*	AAGCAACCCAGCCTTTTCTC	TGAGCATTCTCCTTTGGAC
	Tsp1	*Thbs1*	TAGCTGGAAATGTGGTGCGT	GGCACTTCTTTGCACTCATCG
	Tsp2	*Thbs2*	CAGATTGGCTACGCAGGTGA	GTTTGGGGCAGTTGTCCTTG
	Ucp3	*Ucp3*	CCTACAGAACCATCGCCAGG	ACCGGGGAGGCCACCACTGT
	Vegfr1	*Flt1*	TGGGACAGTAGGAGAGGCTT	GTATTGGTCTGCCGATGGGT
	Vegfr2	*Kdr*	GCGAGACCATTGAAGTGA	GAAGGAGCCAGAAGAACAT
	Wnt5a	*Wnt5a*	TGAATGAACTGGGGGCATCTT	GGCGGTAATTAGGGCTTTCCA

SI Table 3: Antibodies for immunoblotting.

Category	Protein / Target	Host	Dilution	Catalog reference	Source
Primary antibody	*TSP2*	*Rabbit*	1:1000	PA1417	Boster Bio
Primary antibody	*p-eNOS (Ser1179)*	*Mouse*	1:1000	612392	BD Biosciences
Primary antibody	*t-eNOS*	*Mouse*	1:1000	610296	BD Biosciences
Primary antibody	*GAPDH HRP*	*Mouse*	1:10000	ab105428	Abcam
Secondary antibody	*Rabbit*	*Donkey*	1:5000	711-036-152	Jackson Immunoresearch
Secondary antibody	*Mouse*	*Donkey*	1:5000	715-035-150	Jackson Immunoresearch
